# Supplementary material for: The Potential for Effect of a Six-Week Training Program for Gait Aid Use in Older People with Dementia with Unsteadiness of Gait: A Pilot Study
Source: J Clin Med. 2023 Feb 16;12(4):1574. doi: 10.3390/jcm12041574 (PMC9967216; doi:10.3390/jcm12041574)
Supplement: Supplementary file 1 [file jcm-12-01574-s001.zip › Supplemenary Material S2. Gait_aid-training_for_people_with_dementia--2nd_week_second_home_visit.pdf]

Supplementary Material S2

Gait aid-training for people with dementia--2nd week (second home visit)

Start of Block: Demographics--Interview the person with dementia and the caregiver

This is the **second assessment** which occurs at the **second home visit** at the second week.

You will do the following at this home visit:

- Conduct an assessment for the participants before gait aid training (complete first half of the survey)
- Conduct gait aid training with the person with dementia/cognitive impairment
- Conduct an assessment for the participants after gait aid training (complete second half of survey)
- Make your recommendation for home practice and instruct the informal caregiver if applicable. If not applicable, remove the gait aid and bring it back for the next home visit.

This questionnaire includes assessments to be performed **BEFORE and AFTER** the gait aid training session:

Assessments **before** training:

- Number of falls and its associated factors in the last week
- Adverse events in the last week associated with home practice if applicable
- Adherence to home practice sessions carried out by the person with dementia or cognitive impairment and informal caregiver in the last week

Assessment **after** training:

- Rate perception of safety and appropriateness of gait aid use by the physiotherapist, the person with dementia or cognitive impairment and the informal caregiver

---

Please enter the ID of the person with dementia or cognitive impairment (using initials and date of birth). For example John Smith, date of birth 14/2/1950 will be JS14021950

____

Record the date of this home visit. For example 3/1/2021 will be 03012021

____

Did the person with dementia or cognitive impairment fall in the last week?
A fall is defined as "inadvertently coming to rest on the ground, floor or other lower level, excluding intentional change in position to rest in furniture, wall or other objects.

Please enter the number of falls i.e. 0 for no falls, 1 for 1 fall etc. Leave blank if unknown/cannot remember.

____

If a fall happened in the last week, please give details in the item below: (put all information into each box provided respectively if there were multiple falls and/or injuries from falls i.e. format: fall 1 circumstance=xxx, fall 2=xxx etc, injury 1=xxx, injury 2=xxx)

☐ The circumstance of the fall(s) was: (1) ____

☐ The injury from the fall(s) was: enter none if no injury (2) ____

Has the gait aid been left for home practice between sessions?

○ Yes (1)

○ No (2)

If a gait aid was left for home practice, was the person using the gait aid when they fell?

○ Yes. Ask the caregiver if they thought the gait aid might have contributed to the fall--if yes, please give details (1) ____

○ No (2)

○ No fall (3)

Did the person with dementia or cognitive impairment and informal caregiver adhere to home practice in the last week as per the physiotherapist's recommendations?

○ Yes. Full adherence to the frequency and duration of practice (1)

○ No. Partial adherence to the frequency and duration of practice, please state the reasons why. (2) ____

○ No. No practice at all, please state the reasons why. (3) ____

○ No home practice was recommended for the last week (4)

Ask the person with dementia or cognitive impairment:
How did the person with dementia or cognitive impairment find the home practice session (if home practice was applicable)?
Rate on a 0-10 point scale (0=extremely difficult, cannot manage, 10=no difficulties at all, can manage it very well) and provide comments.

____

Ask the informal caregiver:
How did the informal caregiver find the home practice session (if home practice was applicable)?
Rate on a 0-10 point scale (0=extremely difficult, cannot manage, 10=no difficulties at all, can manage it very well) and provide comments.

____

Perform gait aid training with the participant with dementia/cognitive impairment. The following questions are to be answered after gait aid training.

What gait aid have you used to provide training for the person with dementia or cognitive impairment?

- ○ Walking stick/cane (1)
- ○ 4-point stick (2)
- ○ Pick-up frame (3)
- ○ Wheelie walking frame (4)
- ○ Other, please state (5) ____

Has this changed from the previous session?

- ○ Yes. Please explain why and the factors you considered in changing the gait aid. (1) ____
- ○ No (2)

After training--Perception rating of safety and appropriateness of gait aid use.

Ask the **participant** with dementia or cognitive impairment after the gait aid training session to rate the following statements:

	Strongly agree (1)	Somewhat agree (2)	Neither agree nor disagree (3)	Somewhat disagree (4)	Strongly disagree (5)
Walking with (state the type of gait aid) has improved my walking (1)	○	○	○	○	○
Walking with (state the type of gait aid) has made me feel safe (2)	○	○	○	○	○
Walking with (state the type of gait aid) feels right for me (3)	○	○	○	○	○

Ask the **participant with dementia or cognitive impairment** to provide comments for the above responses. Record the comments here.

____

Ask the **informal caregiver** after the gait aid training session to rate the following statements:

	Strongly agree (1)	Somewhat agree (2)	Neither agree nor disagree (3)	Somewhat disagree (4)	Strongly disagree (5)
The gait aid has improved the participant's steadiness in walking (1)	○	○	○	○	○
The gait aid has improved the participant's safety in walking (2)	○	○	○	○	○
Using the gait aid is appropriate for the participant (3)	○	○	○	○	○

Ask the **informal caregiver** to provide comments for the above responses. Record the comments here.

____

Give **your opinion** (i.e. physiotherapist) after the gait aid training session to rate the following statements:

	Strongly agree (1)	Somewhat agree (2)	Neither agree nor disagree (3)	Somewhat disagree (4)	Strongly disagree (5)
The gait aid has improved the participant's steadiness in walking (1)	○	○	○	○	○
The gait aid has improved the participant's safety in walking (2)	○	○	○	○	○
Using the gait aid is appropriate for the participant (3)	○	○	○	○	○

Give your (i.e. physiotherapist) comments for the above responses. Record the comments here.

____

Ask the person with dementia or cognitive impairment:
How did the person with dementia or cognitive impairment find the training session with the physiotherapist?
Rate on a 0-10 point scale (0=extremely difficult, cannot manage, 10=no difficulties at all, can manage it very well) and provide comments.

____

Ask the informal caregiver:
How did the informal caregiver find the training session the person with dementia or cognitive

impairment had with the physiotherapist? Rate on a 0-10 point scale (0=extremely difficult, cannot manage, 10=no difficulties at all, can manage it very well) and provide comments.

____

What recommendations have you provided to the person with dementia or cognitive impairment and their informal caregiver at the end of the session? (based on the responses of the person with dementia/cognitive impairment, objective gait/mobility measurements and your clinical judgement)

○ Leave the gait aid with the person with dementia and informal caregiver to practise at home, please record the instruction here i.e. nature, content, frequency and duration of home practice (1) ____

○ Remove the gait aid at the end of the session (informal caregiver does not feel confident to supervise gait aid practice with the person with dementia). Participant only practices with the physiotherapist. (2)

○ Remove the gait aid at the end of the session (the physiotherapist does not feel it is safe to leave the gait aid for home practice). Participant only practices with the physiotherapist (3)

Please explain the strategies that you have used to help the person with dementia/cognitive impairment to learn the safe and correct use of the gait aid during this session (e.g. memory aids, cues, structuring of practice tasks, environment etc) Select all that apply.

- ☐ Constant pattern of practice (1)
- ☐ Constant pattern of instruction (2)
- ☐ Memory aid (3)
- ☐ Verbal cues (4)
- ☐ Visual cues (5)
- ☐ Start with non-complex environment, then progress to more complex environment (6)
- ☐ Avoid dual tasking during initial learning e.g. minimise talking from the person, talking to the person or stop them from walking with the gait aid when they or you want to talk (7)
- ☐ Other, please provide details (can include the strategies provided by the physiotherapist to the informal caregiver) (8) ____

Do you think the person with dementia/cognitive impairment has successfully demonstrate the safe and correct use of gait aid in this session?

- ○ Yes (1)
- ○ Partially (2)
- ○ No (3)

Do you (physiotherapist) have any further comments regarding the gait aid training program? Please also add the content of your gait aid training session here.

____

Please provide your (physiotherapist) name so that the research team can contact you in case of queries.

____

Thank you for completing the data collection survey. Please move to the next page to submit the survey online.

End of Block: Demographics--Interview the person with dementia and the caregiver
