# Supplementary material for: The Potential for Effect of a Six-Week Training Program for Gait Aid Use in Older People with Dementia with Unsteadiness of Gait: A Pilot Study
Source: J Clin Med. 2023 Feb 16;12(4):1574. doi: 10.3390/jcm12041574 (PMC9967216; doi:10.3390/jcm12041574)
Supplement: Supplementary file 1 [file jcm-12-01574-s001.zip › Supplementary Material S1. Gait_aid-training_for_people_with_dementia--Baseline_first_home_visit.pdf]

Supplementary Material S1

Gait aid-training for people with dementia--Baseline (first home visit)

Start of Block: Demographics--Interview the person with dementia and the caregiver

This is the **baseline assessment** which occurs at the **first home visit.**

You will do the following at this home visit:

- Double check inclusion criteria if required
- Gain consent from the person with dementia/cognitive impairment (apply cognitive capacity checklist) and informal caregiver
- For persons with dementia/cognitive impairment that are unable to give their own consent:
    - For Vic, seek informal caregiver's consent on their behalf. Make sure he/she is a medical decision maker if there is an advanced care directive for this.
    - For WA, please follow the GAA consent procedure.
- Complete the relevant consent form for the person with dementia/cognitive impairment and informal caregiver
- Conduct an assessment for the participants before gait aid training (complete first half of the survey)
- Conduct gait aid training with the person with dementia/cognitive assessment
- Conduct an assessment after gait aid training (complete second half of the survey)
- Make your recommendation for home practice and instruct the informal caregiver if applicable. If not applicable, remove the gait aid and bring it back for the next home visit.
- Give out falls and home practice diary to the participants and instruct them in how to complete them

This questionnaire includes assessments to be performed **BEFORE** and **AFTER** the gait aid training session:

Assessments **before** training:

- Demographics (interview the person with dementia or cognitive impairment and informal caregiver)
- Cognitive assessment (do a cognitive assessment with the person with dementia or cognitive impairment)
- Algorithm items (follow the items in the algorithm for assessment)
- Physical assessments (without a gait aid)

Assessment **after** training:

- Repeat physical assessments (with the recommended gait aid)
- Rate perception of safety and appropriateness of gait aid use by the physiotherapist, the person with dementia or cognitive impairment and the informal caregiver

---

Demographics--Interview the person with dementia or cognitive impairment and informal caregiver.

Please enter the ID of the person with dementia or cognitive impairment (using initials and date of birth). For example John Smith, date of birth 14/2/1950 will be JS14021950

____

Record the date of this home visit. For example 3/1/2021 will be 03012021

____

What is the age of the person with dementia or cognitive impairment? (please enter in years)

____

What is the gender of the person with dementia or cognitive impairment?

○ Male (1)

○ Female (2)

○ Other (3)

○ Prefer not to say (4)

Was the person formally diagnosed with dementia?

○ Yes (1)

○ No (2)

What type of dementia was the person diagnosed with?

- ○ Alzheimer's Disease (1)
- ○ Vascular dementia (2)
- ○ Lewy Body dementia (3)
- ○ Other dementia, please state (4) ____
- ○ Don't know/cannot remember (5)
- ○ The person has not been formally diagnosed with dementia (but meets the protocol definitions of having cognitive impairment) (6)

For how many years has the person been living with dementia (if the person was diagnosed with dementia)? Leave blank if has not been formally diagnosed

____

Who does the person with dementia or cognitive impairment live with? (Select all that apply)

- ▢ Spouse/partner (1)
- ▢ Children (2)
- ▢ Other relatives (3)
- ▢ Friend(s) (4)
- ▢ Lives alone (5)

What medical history does the person with dementia or cognitive impairment have? (Select all that apply)

- ☐ Anxiety (1)
- ☐ Arthritis (2)
- ☐ Cancer (3)
- ☐ Depression (4)
- ☐ Diabetes (5)
- ☐ Heart disease (6)
- ☐ Hip joint replacement (7)
- ☐ Kidney disease/renal failure (8)
- ☐ Knee joint replacement (9)
- ☐ Lung disease/respiratory disease (10)
- ☐ Parkinson's disease (11)
- ☐ Stroke (12)
- ☐ Other neurological disease (13)
- ☐ Visual impairment that are not correctable by glasses e.g. macular degeneration, glaucoma (14)
- ☐ Other, please state (15) ____

Physiotherapist to answer this question:
Can the person with dementia or cognitive impairment move around their home, including stopping and turning (observe)?

- can do, appears steady (1)
- can do but appears unsteady (2)
- cannot do without assistance from someone else (3)

Can the person with dementia or cognitive impairment move outside their home (self i.e. person with dementia/cognitive impairment or carer report)?

- can do, reported as steady and safe (1)
- can do but reported as unsteady or unsafe (2)
- cannot do without assistance from someone else (3)

Can the person with dementia or cognitive impairment walk up and down at least 3 stairs without a handrail or assistance from someone else (self i.e. person with dementia/cognitive impairment or carer report)?

- can do without difficulty (1)
- can do but with difficulty, or needs rail assistance (2)
- cannot do without assistance from someone else (3)

Physiotherapist to answer this question:
Can the person with dementia or cognitive impairment bend and pick up an object from the floor without assistance from someone else (observe)?

- ○ can do without difficulty (1)
- ○ can do but with difficulty or unsteady (2)
- ○ cannot do without assistance from someone else (3)

Is the person with dementia or cognitive impairment currently engaging in a regular exercise or a physical activity program?

- ○ Yes, please describe what it is and how often, and overall exercise duration (minutes) for the past week (1) ____
- ○ No (2)

ALGORITHM ITEMS

Cognitive assessment:

Use cognitive test provided (Rowland Universal Dementia Assessment Scale-RUDAS) to assess the person with dementia or cognitive impairment.
N.B: Severity of cognitive impairment (RUDAS) 17-22=mild severity, 10-16= moderate severity, less than 10=severe dementia

The total score from the cognitive assessment is:

○ Mild severity, and the score is: (4)

___

○ Moderate severity, and the score is: (5)

___

○ Severe dementia, and the score is: (6)

___

Self (or carer's) report on ability of the person with dementia/cognitive impairment to remember new information

○ Can remember some information day to day (1)

○ Can remember some information over time, but limited day to day (2)

○ Cannot remember new information day to day (3)

Self (or carer's) rating of steadiness in walking, turning, going up and down stairs

○ Mostly steady, only occasional unsteadiness in turning or going up and down stairs (1)

○ Some unsteadiness on 1 to 2 of these activities (2)

○ Unsteady on all activities (3)

Did the person with dementia or cognitive impairment fall in the past 12 months?
A fall is defined as inadvertently coming to rest on the ground, floor or other lower level,

excluding intentional change in position to rest in furniture, wall or other objects.

- ○ No falls (4)
- ○ 1-3 falls (5)
- ○ 4 or more falls (6)

Score the Elderly Falls Screening Tool (EFST). Refer to the training manual for scoring instructions. The EFST score is:

____

Perform the TUG test (at a comfortable speed) without a gait aid. Allow a practice trial before the test.
Ask the person with dementia or cognitive impairment to stand up from a standard height (46cm) kitchen chair, walk three metres at their comfortable speed, turn, and return to the chair and sit down.

The time required (in seconds, to one decimal place) is:

____

Perform a walking test in a straight path (choose a corridor in the participant's home), then repeat the test asking the participant with dementia/cognitive impairment to count aloud backwards by 1's from 50 concurrently while walking.

- ○ Able to walk and count aloud without slowing or stopping (1)
- ○ Slows down when counting aloud but can keep walking (2)
- ○ Unable to walk and counting aloud concurrently (i.e. needs to stop) (3)

Has the participant with dementia/cognitive impairment been taught by a physiotherapist in how to use a gait aid in the past and was it successful?

- ○ No (1)
- ○ Yes, was successful but the participant did not persist with gait aid use (2)
- ○ Yes but was unsuccessful (3)

Before training--Physical assessment: 4 metre walk test

Perform the 4 metre walk test (at a comfortable walking speed) **without a gait aid.** Allow a practice trial before the test.

- ☐ Time required (in seconds, to one decimal place) (1) ________________________________
- ☐ Gait speed (metre per second i.e. 4 divided by the time taken) (2) ________________________________
- ☐ No. of steps (3) ________________________________
- ☐ Step length (i.e. 4 m divided by the no. of steps) (4) ________________________________

Before training--Physical assessment: Figure of 8 walkway

Perform the Figure of 8 walkway test (at a comfortable speed) **without a gait aid.** Allow a practice trial before the test.

Instructions to the person with dementia or cognitive impairment:

- Stand between the 2 cones (placed 5 feet apart i.e. 1.5 m), facing in one direction (the starting/stopping point is not marked "… in order to avoid influencing the movement planning for the task.")
- Begin walking at your usual pace when ready (the person chooses which direction to go around the cones)
- Stop when you return to the start position.

Instructions to the therapist:

- Timing: begin timing with the first step, and stop timing when the person with dementia assumes a side-by-side stance of the feet back at the start position.
- Accuracy: "The 2-ft (0.6 m) surround test boundary was not marked on the course. The tester determined the 2-ft (0.6 m) boundary area for the test setup and the relationships of the boundary to the testing space (e.g. distance from the hallway walls, floor markings, or landmarks) prior to testing, and estimated whether the test was completed within the boundary by comparison with the tester's mental map of the testing space."

☐ Time required (in seconds, to one decimal place) (1)

____

☐ Accuracy: does the person stay within the unmarked 2-foot (0.6 m) surround boundary of the cones? Write Yes or No response only. (2)

____

Before training-repeat the Figure of 8 walkway test (at a comfortable speed) **without a gait aid AND with a dual cognitive task.** Allow a practice trial before the test.

Instructions to the person with dementia or cognitive impairment:

- Stand between the 2 cones (placed 5 feet apart i.e. 1.5 m), facing in one direction (the starting/stopping point is not marked "… in order to avoid influencing the movement planning for the task.")
- Begin walking at your usual pace when ready (the person chooses which direction to go around the cones) and count backwards by 1's from 50 whilst walking.
- Stop when you return to the start position.

Instructions to the therapist:

- Timing: begin timing with the first step, and stop timing when the person with dementia assumes a side-by-side stance of the feet back at the start position.
- Accuracy: "The 2-ft (0.6 m) surround test boundary was not marked on the course. The tester determined the 2-ft (0.6 m) boundary area for the test setup and the relationships of the boundary to the testing space (e.g. distance from the hallway walls, floor

markings, or landmarks) prior to testing, and estimated whether the test was completed within the boundary by comparison with the tester's mental map of the testing space."

☐ Time required (in seconds, to one decimal place) (1)

____

☐ Accuracy: does the person stay within the unmarked 2-foot (0.6 m) surround boundary of the cones? Write Yes or No response only. (2)

____

☐ Counting errors: Write 1=Only 0 or 1 are incorrect responses, 2=Only 2 or 3 are incorrect responses, 3=Only 2 or 3 are correct responses, 4=Only 0 or 1 are correct responses, 5=Needs to stop (unable to walk and count at the same time). (3)

____

Having completed this assessment (before starting gait aid training) – what is your (the physiotherapist) perception of the likelihood of this person with dementia/cognitive impairment being able to achieve safe gait aid training at the end of this 6 week training? (0=extremely unlikely, 5=moderate likely and 10=extremely likely)

	0 (1)	1 (2)	2 (3)	3 (4)	4 (5)	5 (6)	6 (7)	7 (8)	8 (9)	9 (10)	10 (11)
Perception of likelihood of the person being able to achieve safe gait aid training at the end of the 6 week training program (1)	○	○	○	○	○	○	○	○	○	○	○

Do you consider it safe and acceptable to the person with dementia and their caregiver to proceed with the 6 week gait aid training program?

○ Yes. Proceed to the next question (1)

○ No. Finish here, thank the person with dementia/cognitive impairment and their carer for their time. Clarify for them the reasons you will not be proceeding with the gait aid training program. State the reason why. (2) ____

Perform gait aid training with the participant with dementia/cognitive impairment.

The following questions are to be answered after the gait aid training session.

What gait aid have you used to provide training for the person with dementia or cognitive impairment?

○ Walking stick/cane (1)

○ 4-point stick (2)

○ Pick-up frame (3)

○ Wheelie walking frame (4)

○ Other, please state (5) ____

State the main reason(s) for selecting this gait aid.

____

After training--Perception rating of safety and appropriateness of gait aid use.

Ask the **participant** with dementia or cognitive impairment after the gait aid training session to rate the following statements:

	Strongly agree (1)	Somewhat agree (2)	Neither agree nor disagree (3)	Somewhat disagree (4)	Strongly disagree (5)
Walking with (state the type of gait aid) has improved my walking (1)	○	○	○	○	○
Walking with (state the type of gait aid) has made me feel safe (2)	○	○	○	○	○
Walking with (state the type of gait aid) feels right for me (3)	○	○	○	○	○

Ask the **participant with dementia or cognitive impairment** to provide comments for the above responses. Record the comments here.

____

Ask the **informal caregiver** after the gait aid training session to rate the following statements:

	Strongly agree (1)	Somewhat agree (2)	Neither agree nor disagree (3)	Somewhat disagree (4)	Strongly disagree (5)
The gait aid has improved the participant's steadiness in walking (1)	○	○	○	○	○
The gait aid has improved the participant's safety in walking (2)	○	○	○	○	○
Using the gait aid is appropriate for the participant (3)	○	○	○	○	○

Ask the **informal caregiver** to provide comments for the above responses. Record the comments here.

____

Give **your opinion (i.e. physiotherapist)** after the gait aid training session to rate the following statements:

	Strongly agree (1)	Somewhat agree (2)	Neither agree nor disagree (3)	Somewhat disagree (4)	Strongly disagree (5)
The gait aid has improved the participant's steadiness in walking (1)	○	○	○	○	○
The gait aid has improved the participant's safety in walking (2)	○	○	○	○	○
Using the gait aid is appropriate for the participant (3)	○	○	○	○	○

Give your (i.e. physiotherapist) comments for the above responses. Record the comments here.

____

After training--Physical assessment: 4 metre walk test

Perform the 4 metre walk test (at a comfortable walking speed) **with the recommended gait aid.** Allow a practice trial before the test.

- [ ] Time required (in seconds, to one decimal place) (1) ________________________________

- [ ] Gait speed (metre per second i.e. 4 divided by the time taken) (2) ________________________________

- [ ] No. of steps (3) ________________________________

- [ ] Step length (i.e. 4 m divided by the no. of steps) (4) ________________________________

After training--Physical assessment: Timed Up and Go (TUG) Test

Perform the TUG test (at a comfortable speed) **with the recommended gait aid**. Allow a practice trial before the test.

Ask the person with dementia or cognitive impairment to stand up from a standard height kitchen chair (46cm), walk three metres at their comfortable speed, turn, and return to the chair and sit down.

- [ ] Time required (in seconds, to one decimal place) (1) ________________________________

After training--Physical assessment: Figure of 8 walkway

Perform the Figure of 8 walkway test (at a comfortable speed) **with the recommended gait aid.** Allow a practice trial before the test.

Instructions to person with dementia or cognitive impairment:

- Stand between the 2 cones (placed 5 feet apart i.e. 1.5 m), facing in one direction (the starting/stopping point is not marked "… in order to avoid influencing the movement planning for the task.")
- Begin walking at your <u>usual pace</u> when ready (the person chooses which direction to go around the cones)
- Stop when you return to the start position.

Instructions to the therapist:

- Timing: begin timing with the first step, and stop timing when the person with dementia assumes a side-by-side stance of the feet back at the start position.
- Accuracy: "The 2-ft (0.6 m) surround test boundary was not marked on the course. The tester determined the 2-ft (0.6 m) boundary area for the test setup and the relationships of the boundary to the testing space (e.g. distance from the hallway walls, floor markings, or landmarks) prior to testing, and estimated whether the test was completed within the boundary by comparison with the tester's mental map of the testing space."

☐ Time required (in seconds, to one decimal place) (1)

____

☐ Accuracy: does the person stay within the unmarked 2-foot (0.6 m) surround boundary of the cones? Write Yes or No response only. (2)

____

After training-repeat the Figure of 8 walkway test (at a comfortable speed) **with the recommended gait aid AND with a dual cognitive task.** Allow a practice trial before the test.

Instructions to person with dementia or cognitive impairment:

- Stand between the 2 cones (placed 5 feet apart i.e. 1.5 m), facing in one direction (the starting/stopping point is not marked "… in order to avoid influencing the movement planning for the task.")
- Begin walking at your <u>usual pace</u> when ready (the person chooses which direction to go around the cones) and count backwards by 1's from 50 whilst walking.
- Stop when you return to the start position.

Instructions to the therapist:

- Timing: begin timing with the first step, and stop timing when the person with dementia assumes a side-by-side stance of the feet back at the start position.
- Accuracy: "The 2-ft (0.6 m) surround test boundary was not marked on the course. The tester determined the 2-ft (0.6 m) boundary area for the test setup and the relationships

of the boundary to the testing space (e.g. distance from the hallway walls, floor markings, or landmarks) prior to testing, and estimated whether the test was completed within the boundary by comparison with the tester's mental map of the testing space."

☐ Time required (in seconds, to one decimal place) (1)

____

☐ Accuracy: does the person stay within the unmarked 2-foot (0.6 m) surround boundary of the cones? Write Yes or No response only. (2)

____

☐ Counting errors: Write 1=Only 0 or 1 are incorrect responses, 2=Only 2 or 3 are incorrect responses, 3=Only 2 or 3 are correct responses, 4=Only 0 or 1 are correct responses, 5=Needs to stop (unable to walk and count at the same time). (3)

____

Ask the person with dementia or cognitive impairment:
How did the person with dementia or cognitive impairment find the training session with the physiotherapist?
Rate on a 0-10 point scale (0=extremely difficult, cannot manage, 10=no difficulties at all, can manage it very well) and provide comments.

____

Ask the informal caregiver:
How did the informal caregiver find the training session the person with dementia or cognitive impairment had with the physiotherapist?
Rate on a 0-10 point scale (0=extremely difficult, cannot manage, 10=no difficulties at all, can manage it very well) and provide comments.

____

What recommendations have you provided to the person with dementia or cognitive impairment and their informal caregiver at the end of the session? (based on the responses of the person

with dementia/cognitive impairment, objective gait/mobility measurements and your clinical judgement)

- ○ Leave the gait aid with the person with dementia and informal caregiver to practise at home, please record the instruction here i.e. nature, content, frequency and duration of home practice (1) ____

- ○ Remove the gait aid at the end of the session (informal caregiver does not feel confident to supervise gait aid practice with the person with dementia). Participant only practices with the physiotherapist. (2)

- ○ Remove the gait aid at the end of the session (the physiotherapist does not feel it is safe to leave the gait aid for home practice). Participant only practices with the physiotherapist. (3)

Please explain the strategies that you have used to help the person with dementia/cognitive impairment to learn the safe and correct use of the gait aid during this session (e.g. memory aids, cues, structuring of practice tasks, environment etc) Select all that apply.

- ▢ Constant pattern of practice (1)
- ▢ Constant pattern of instruction (2)
- ▢ Memory aid (3)
- ▢ Verbal cues (4)
- ▢ Visual cues (5)
- ▢ Start with non-complex environment, then progress to more complex environment (6)
- ▢ Avoid dual tasking during initial learning e.g. minimise talking from the person, talking to the person or stop them from walking with the gait aid when they or you want to talk (7)
- ▢ Other, please provide details (can include the strategies provided by the physiotherapist to the informal caregiver) (8) ____

Do you think the person with dementia/cognitive impairment has successfully demonstrated the safe and correct use of gait aid in this session?

- ○ Yes (1)
- ○ Partially (2)
- ○ No (3)

Do you (physiotherapist) have any further comments regarding the gait aid training program? Please also add the content of your gait aid training session here.

____

Please provide your (physiotherapist) name so that the research team can contact you in case of queries.

____

Thank you for completing the data collection survey. Please move to the next page to submit the survey online.

End of Block: Demographics--Interview the person with dementia and the caregiver
