# Supplementary material for: The Potential for Effect of a Six-Week Training Program for Gait Aid Use in Older People with Dementia with Unsteadiness of Gait: A Pilot Study
Source: J Clin Med. 2023 Feb 16;12(4):1574. doi: 10.3390/jcm12041574 (PMC9967216; doi:10.3390/jcm12041574)
Supplement: Supplementary file 1 [file jcm-12-01574-s001.zip › Supplementary Material S4.Gait_aid-training_for_people_with_dementia--6th_week_fourth_home_visit.pdf]

Supplementary Material S4

Gait aid-training for people with dementia--6th week (fourth home visit)

Start of Block: Demographics--Interview the person with dementia and the caregiver

This is the **final assessment (for the participants who you do not recommend use of gait aid after this session)** which occurs at the **fourth home visit** at the 6th week.

You will do the following at this home visit:

- Conduct an assessment for the participants before gait aid training (complete first half of the survey)
- Conduct gait aid training with the person with dementia/cognitive impairment
- Conduct an assessment for the participants after gait aid training (complete second half of survey)
- Make your recommendation for whether the participants will continue to use the gait aid in the longer term or not. If you do not recommend gait aid use in the longer term, remove the gait aid from the participant.
- Collect falls and home practice diary from participants who you do not recommend use of gait aid after this session OR
- Collect home practice diary only from participants who you recommend long term use of gait aid after this session and instruct them to continue filling in their falls diary

This questionnaire includes assessments to be performed **BEFORE and AFTER** the gait aid training session:

Assessments **before** training:

- Number of falls and its associated factors in the last two weeks
- Adverse events in the last two weeks associated with home practice if applicable
- Adherence to home practice sessions carried out by the person with dementia or cognitive impairment and informal caregiver in the last two weeks
- Physical assessments (without a gait aid)

Assessment **after** training:

- Repeat physical assessments (with the recommended gait aid)
- Rate perception of safety and appropriateness of gait aid use by the physiotherapist, the person with dementia or cognitive impairment and the informal caregiver

---

Please enter the ID of the person with dementia or cognitive impairment (using initials and date of birth). For example John Smith, date of birth 14/2/1950 will be JS14021950

____

Record the date of this home visit. For example 3/1/2021 will be 03012021

____

Physiotherapist to answer this question:
Can the person with dementia or cognitive impairment move around their home, including

stopping and turning (observe with gait aid if it has been left with the person, or without if it has not been left with the person)?

- ○ can do, appears steady (without gait aid) (1)
- ○ can do but appears unsteady (without gait aid) (2)
- ○ cannot do without assistance from someone else (without gait aid) (3)
- ○ can do, appears steady (with gait aid) (4)
- ○ can do but appears unsteady (with gait aid) (5)
- ○ cannot do without assistance from someone else (with gait aid) (6)

Can the person with dementia or cognitive impairment move outside their home (self i.e. person with dementia/cognitive impairment or carer report)?

- ○ can do, reported as steady and safe (without gait aid) (1)
- ○ can do but reported as unsteady or unsafe (without gait aid) (2)
- ○ cannot do without assistance from someone else (without gait aid) (3)
- ○ can do, reported as steady and safe (with gait aid) (4)
- ○ can do but reported as unsteady or unsafe (with gait aid) (5)
- ○ cannot do without assistance from someone else (with gait aid) (6)

Can the person with dementia or cognitive impairment walk up and down at least 3 stairs without a handrail or assistance from someone else (self i.e. person with dementia/cognitive impairment or carer report)?

○ can do without difficulty (without gait aid) (1)

○ can do but with difficulty, or needs rail assistance (without gait aid) (2)

○ cannot do without assistance from someone else (without gait aid) (3)

○ can do without difficulty (with gait aid-only score for unilateral gait aid) (4)

○ can do but with difficulty, or needs rail assistance (with gait aid-only score for unilateral gait aid) (5)

○ cannot do without assistance from someone else (with gait aid-only score for unilateral gait aid) (6)

Physiotherapist to answer this question:
Can the person with dementia or cognitive impairment bend and pick up an object from the floor without assistance from someone else (observe)?

○ can do without difficulty (1)

○ can do but with difficulty or unsteady (2)

○ cannot do without assistance from someone else (3)

Did the person with dementia or cognitive impairment fall in the last 2 weeks?
A fall is defined as "inadvertently coming to rest on the ground, floor or other lower level, excluding intentional change in position to rest in furniture, wall or other objects.

Please enter the number of falls i.e. 0 for no falls, 1 for 1 fall etc. Leave blank if unknown/cannot remember.

____

If a fall happened in the last two weeks, please give details in the item below: (put all information into each box provided respectively if there were multiple falls and/or injuries from falls i.e. format: fall 1 circumstance=xxx, fall 2=xxx etc, injury 1=xxx, injury 2=xxx)

- ☐ The circumstance of the fall(s) was: (1) ____

- ☐ The injury from the fall(s) was: enter none if no injury (2) ____

Has the gait aid been left for home practice between sessions?

- ○ Yes (1)
- ○ No (2)

If a gait aid was left for home practice, was the person using the gait aid when they fell?

- ○ Yes. Ask the caregiver if they consider that the gait aid might have contributing to the fall--if yes, please give details (1) ____
- ○ No (2)
- ○ No fall (3)

Did the person with dementia or cognitive impairment and informal caregiver adhere to home practice in the last 2 weeks as per the physiotherapist's recommendations?

○ Yes. Full adherence to the frequency and duration of practice (1)

○ No. Partial adherence to the frequency and duration of practice, please state the reasons why. (2) ____

○ No. No practice at all, please state the reasons why. (3)
____

○ No home practice was recommended for the last 2 weeks (4)

Ask the person with dementia or cognitive impairment: How did the person with dementia or cognitive impairment find the home practice session (if home practice was applicable)? Rate on a 0-10 point scale (0=extremely difficult, cannot manage, 10=no difficulties at all, can manage it very well) and provide comments.

____

Ask the informal caregiver: How did the informal caregiver find the home practice session (if home practice was applicable)? Rate on a 0-10 point scale (0=extremely difficult, cannot manage, 10=no difficulties at all, can manage it very well) and provide comments.

____

Before training--Physical assessment: 4 metre walk test

Perform the 4 metre walk test (at a comfortable walking speed) **without a gait aid.** Allow a practice trial before the test.

- [ ] Time required (in seconds, to one decimal place) (1) ______________________________

- [ ] Gait speed (metre per second i.e. 4 divided by the time taken) (2) ______________________________

- [ ] No. of steps (3) ______________________________

- [ ] Step length (i.e. 4 m divided by the no. of steps) (4) ______________________________

Before training--Physical assessment: Figure of 8 walkway

Perform the Figure of 8 walkway test (at a comfortable speed) **without a gait aid.** Allow a practice trial before the test.

Instructions to the person with dementia or cognitive impairment:

- Stand between the 2 cones (placed 5 feet apart i.e. 1.5 m), facing in one direction (the starting/stopping point is not marked "… in order to avoid influencing the movement planning for the task.")
- Begin walking at your <u>usual pace</u> when ready (the person chooses which direction to go around the cones)
- Stop when you return to the start position.

Instructions to the therapist:

- Timing: begin timing with the first step, and stop timing when the person with dementia assumes a side-by-side stance of the feet back at the start position.
- Accuracy: "The 2-ft (0.6 m) surround test boundary was not marked on the course. The tester determined the 2-ft (0.6 m) boundary area for the test setup and the relationships of the boundary to the testing space (e.g. distance from the hallway walls, floor

markings, or landmarks) prior to testing, and estimated whether the test was completed within the boundary by comparison with the tester’s mental map of the testing space.”

☐ Time required (in seconds, to one decimal place) (1)

____

☐ Accuracy: does the person stay within the unmarked 2-foot (0.6 m) surround boundary of the cones? Write Yes or No response only (2)

____

Before training--repeat the Figure of 8 walkway test (at a comfortable speed) **without a gait aid AND with a dual cognitive task.** Allow a practice trial before the test.

Instructions to the person with dementia or **cognitive** impairment:

- Stand between the 2 cones (placed 5 feet apart i.e. 1.5 m), facing in one direction (the starting/stopping point is not marked “… in order to avoid influencing the movement planning for the task.”)
- Begin walking at your usual pace when ready (the person chooses which direction to go around the cones) and count backwards by 1's from 50 whilst walking.
- Stop when you return to the start position.

Instructions to the therapist:

- Timing: begin timing with the first step, and stop timing when the person with dementia assumes a side-by-side stance of the feet back at the start position.
- Accuracy: “The 2-ft (0.6 m) surround test boundary was not marked on the course. The tester determined the 2-ft (0.6 m) boundary area for the test setup and the relationships of the boundary to the testing space (e.g. distance from the hallway walls, floor

markings, or landmarks) prior to testing, and estimated whether the test was completed within the boundary by comparison with the tester's mental map of the testing space."

☐ Time required (in seconds, to one decimal place) (1)

____

☐ Accuracy: does the person stay within the unmarked 2-foot (0.6 m) surround boundary of the cones? Write Yes or No response only. (2)

____

☐ Counting errors: Write 1=Only 0 or 1 are incorrect responses, 2=Only 2 or 3 are incorrect responses, 3=Only 2 or 3 are correct responses, 4=Only 0 or 1 are correct responses, 5=Needs to stop (unable to walk and count at the same time). (3)

____

Perform gait aid training with the participant with dementia/cognitive impairment. The following questions are to be answered after gait aid training.

What gait aid have you used to provide training for the person with dementia or cognitive impairment?

- ○ Walking stick/cane (1)
- ○ 4-point stick (2)
- ○ Pick-up frame (3)
- ○ Wheelie walking frame (4)
- ○ Other, please state (5) ________________________________

Has this changed from the previous session?

○ Yes. Please explain why and the factors you considered in changing the gait aid. (1) ____

○ No (2)

After training--Perception rating of safety and appropriateness of gait aid use.

Ask the **participant** with dementia or cognitive impairment after the gait aid training session to rate the following statements:

	Strongly agree (1)	Somewhat agree (2)	Neither agree nor disagree (3)	Somewhat disagree (4)	Strongly disagree (5)
Walking with (state the type of gait aid) has improved my walking (1)	○	○	○	○	○
Walking with (state the type of gait aid) has made me feel safe (2)	○	○	○	○	○
Walking with (state the type of gait aid) feels right for me (3)	○	○	○	○	○

Ask the **participant with dementia or cognitive impairment** to provide comments for the above responses. Record the comments here.

____

Ask the **informal caregiver** after the gait aid training session to rate the following statements:

	Strongly agree (1)	Somewhat agree (2)	Neither agree nor disagree (3)	Somewhat disagree (4)	Strongly disagree (5)
The gait aid has improved the participant's steadiness in walking (1)	○	○	○	○	○
The gait aid has improved the participant's safety in walking (2)	○	○	○	○	○
Using the gait aid is appropriate for the participant (3)	○	○	○	○	○

Ask the **informal caregiver** to provide comments for the above responses. Record the comments here.

____

Give **your opinion** (i.e. physiotherapist) after the gait aid training session to rate the following statements:

	Strongly agree (1)	Somewhat agree (2)	Neither agree nor disagree (3)	Somewhat disagree (4)	Strongly disagree (5)
The gait aid has improved the participant's steadiness in walking (1)	○	○	○	○	○
The gait aid has improved the participant's safety in walking (2)	○	○	○	○	○
Using the gait aid is appropriate for the participant (3)	○	○	○	○	○

Give your (i.e. physiotherapist) comments for the above responses. Record the comments here.

____

After training--Physical assessment: 4 metre walk test

Perform the 4 metre walk test (at a comfortable walking speed) **with the recommended gait aid.** Allow a practice trial before the test.

☐ Time required (in seconds, to one decimal place) (1)

____

☐ Gait speed (metre per second i.e. 4 divided by the time taken) (2)

____

☐ No. of steps (3) ____

☐ Step length (i.e. 4 m divided by the no. of steps) (4)

____

After training--Physical assessment: Timed Up and Go (TUG) Test

Perform the TUG test (at a comfortable speed) **with the recommended gait aid.** Allow a practice trial before the test.

Ask the person with dementia or cognitive impairment to stand up from a standard height kitchen chair (46cm), walk three metres at their comfortable speed, turn, and return to the chair and sit down.

☐ Time required (in seconds, to one decimal place) (1)

____

After training--Physical assessment: Figure of 8 walkway

Perform the Figure of 8 walkway test (at a comfortable speed) **with the recommended gait aid.** Allow a practice trial before the test.

Instructions to the person with dementia or cognitive impairment:

- Stand between the 2 cones (placed 5 feet apart i.e. 1.5 m), facing in one direction (the starting/stopping point is not marked "… in order to avoid influencing the movement planning for the task.")
- Begin walking at your <u>usual pace</u> when ready (the person chooses which direction to go around the cones)
- Stop when you return to the start position.

Instructions to the therapist:

- Timing: begin timing with the first step, and stop timing when the person with dementia assumes a side-by-side stance of the feet back at the start position.
- Accuracy: "The 2-ft (0.6 m) surround test boundary was not marked on the course. The tester determined the 2-ft (0.6 m) boundary area for the test setup and the relationships of the boundary to the testing space (e.g. distance from the hallway walls, floor markings, or landmarks) prior to testing, and estimated whether the test was completed within the boundary by comparison with the tester's mental map of the testing space."

☐ Time required (in seconds, to one decimal place) (1)

____

☐ Accuracy: does the person stay within the unmarked 2-foot (0.6 m) surround boundary of the cones? Write Yes or No response only. (2)

____

After training--repeat the Figure of 8 walkway test (at a comfortable speed) **with the recommended gait aid AND with a dual cognitive task.** Allow a practice trial before the test.

Instructions to the person with dementia or cognitive impairment:

- Stand between the 2 cones (placed 5 feet apart i.e. 1.5 m), facing in one direction (the starting/stopping point is not marked "… in order to avoid influencing the movement planning for the task.")
- Begin walking at your <u>usual pace</u> when ready (the person chooses which direction to go around the cones) and count backwards by 1's from 50 whilst walking.
- Stop when you return to the start position.

Instructions to the therapist:

- Timing: begin timing with the first step, and stop timing when the person with dementia assumes a side-by-side stance of the feet back at the start position.
- Accuracy: "The 2-ft (0.6 m) surround test boundary was not marked on the course. The tester determined the 2-ft (0.6 m) boundary area for the test setup and the relationships of the boundary to the testing space (e.g. distance from the hallway walls, floor

markings, or landmarks) prior to testing, and estimated whether the test was completed within the boundary by comparison with the tester's mental map of the testing space."

- [ ] Time required (in seconds, to one decimal place) (1)

____

- [ ] Accuracy: does the person stay within the unmarked 2-foot (0.6 m) surround boundary of the cones? Write Yes or No response only. (2)

____

- [ ] Counting errors: Write 1=Only 0 or 1 are incorrect responses, 2=Only 2 or 3 are incorrect responses, 3=Only 2 or 3 are correct responses, 4=Only 0 or 1 are correct responses, 5=Needs to stop (unable to walk and count at the same time). (3)

____

Ask the person with dementia or cognitive impairment: How did the person with dementia or cognitive impairment find the training session with the physiotherapist? Rate on a 0-10 point scale (0=extremely difficult, cannot manage, 10=no difficulties at all, can manage it very well) and provide comments.

____

Ask the informal caregiver:
How did the informal caregiver find the training session the person with dementia or cognitive impairment had with the physiotherapist?
Rate on a 0-10 point scale (0=extremely difficult, cannot manage, 10=no difficulties at all, can manage it very well) and provide comments.

____

What recommendations have you provided to the person with dementia or cognitive impairment and their informal caregiver at the end of the session? (based on the responses of the person with dementia/cognitive impairment and objective gait/mobility measurements)

- ○ Leave the gait aid with the person with dementia and informal caregiver to use in the long term at all times when up and ambulating. (1)
- ○ Leave the gait aid with the person with dementia and informal caregiver to use in the long term but only at some of the time, please state the situations when the person is recommended to use the gait aid. (2) ____
- ○ Remove the gait aid at the end of the session. Do not recommend the person with dementia to use gait aid. (3)

Please explain the strategies that you have used to help the person with dementia/cognitive impairment to learn the safe and correct use of the gait aid during this session (e.g. memory aids, cues, structuring of practice tasks, environment etc) Select all that apply.

- ☐ Constant pattern of practice (1)
- ☐ Constant pattern of instruction (2)
- ☐ Memory aid (3)
- ☐ Verbal cues (4)
- ☐ Visual cues (5)
- ☐ Start with non-complex environment, then progress to more complex environment (6)
- ☐ Avoid dual tasking during initial learning e.g. minimise talking from the person, talking to the person or stop them from walking with the gait aid when they or you want to talk (7)
- ☐ Other, please provide details (can include the strategies provided by the physiotherapist to the informal caregiver) (8) ____

Do you think the person with dementia/cognitive impairment has successfully demonstrate the safe and correct use of gait aid in this session?

- ○ Yes (1)
- ○ Partially (2)
- ○ No (3)

Has this person with dementia or cognitive impairment successfully achieved safe gait aid use at the completion of the 6-week training program?

○ Yes (1)

○ No (2)

Do you think the length of the training program is adequate for the person with dementia/cognitive impairment to learn the safe and correct use of the recommended gait aid?

○ Yes. Please suggest the length of training you think the person actually needed and explain why (including if less than 6 weeks). (1)
____

○ No. Please suggest how much longer (i.e. on top of 6 weeks) you think the person will need and explain why. (2) ____

○ Unsure. Length of the training program is not the primary issue and explain why. (3)
____

Do you think the frequency of home visits is adequate for the person with dementia/cognitive impairment to learn the safe and correct use of the recommended gait aid?

○ Yes. Please explain why. (1)
____

○ No. Please suggest how frequent the home visits will need to be and explain why. (2)
____

○ Unsure. Frequency of home visits is not the primary issue and explain why. (3)
____

Are there any changes to the standard training program you would like to make? (based on the responses of the person with dementia/cognitive impairment and objective gait/mobility measurements)

____

Do you (physiotherapist) have any further comments regarding the gait aid training program? Please also add the content of your gait aid training session here.

____

Please provide your (physiotherapist) name so that the research team can contact you in case of queries.

____

Thank you for completing the data collection survey. Please move to the next page to submit the survey online.

End of Block: Demographics--Interview the person with dementia and the caregiver
