# Supplementary material for: The Potential for Effect of a Six-Week Training Program for Gait Aid Use in Older People with Dementia with Unsteadiness of Gait: A Pilot Study
Source: J Clin Med. 2023 Feb 16;12(4):1574. doi: 10.3390/jcm12041574 (PMC9967216; doi:10.3390/jcm12041574)
Supplement: Supplementary file 1 [file jcm-12-01574-s001.zip › Supplementary Material S5.Gait_aid-training_for_people_with_dementia--6_weeks_follow-up_post_program.pdf]

Supplementary Material S5

Gait aid-training for people with dementia--6 weeks follow-up post program

---

Start of Block: Interview the person with dementia and the caregiver

This is the **6 week follow-up assessment** post program for the participants who you recommended to continue using the gait aid after the gait aid training program.

You will do the following at this follow-up:

- Call the participant to ask if they have continued to use the gait aid in the last 6 weeks. If so, arrange for a home visit. If they have stopped using the gait aid completely, then complete the first half of the survey only on the phone (No home visit is required except to collect the items below).
- Collect the gait aid and falls diary from the participant

For the participants who require a home visit:

- No gait aid training is required in this follow-up
- Conduct an assessment with the participants (complete all parts of the survey)
- Discuss with the participants regarding recommendation of long term use of the gait aid or not.
- Collect the falls diary from the participants.

Please enter the ID of the person with dementia or cognitive impairment (using initials and date of birth). For example John Smith, date of birth 14/2/1950 will be JS14021950

____

Record the date of this assessment. For example 3/1/2021 will be 03012021

____

Did the person with dementia or cognitive impairment fall in the last 6 weeks?
A fall is defined as "inadvertently coming to rest on the ground, floor or other lower level, excluding intentional change in position to rest in furniture, wall or other objects.

Please enter the number of falls i.e. 0 for no falls, 1 for 1 fall etc. Leave blank if unknown/cannot remember.

____

If a fall happened in the last 6 weeks, please give details in the item below:(put all information into each box provided respectively if there were multiple falls and/or injuries from falls i.e. format: fall 1 circumstance=xxx, fall 2=xxx etc, injury 1=xxx, injury 2=xxx)

- ☐ The circumstance of the fall(s) was: (1) ____
- ☐ The injury from the fall(s) was: enter none if no injury (2) ____

Was the person using the gait aid when they fell?

- ○ Yes. Ask the caregiver if they thought the gait aid might have contributed to the fall--if yes, please give details (1) ____
- ○ No (2)
- ○ No fall (3)
- ○ No, participant has stopped using the gait aid (4)

What gait aid have you recommended for the person with dementia or cognitive impairment?

- ○ Walking stick/cane (1)
- ○ 4-point stick (2)
- ○ Pick-up frame (3)
- ○ Wheelie walking frame (4)
- ○ Other, please state (5) ____

Has the person with dementia or cognitive impairment continued to use the recommended gait aid?

- ○ Yes. At all times when walking. Continue with the rest of the survey (1)
- ○ Partial use. Only for certain tasks or at times. Please estimate the percentage of the time walking that the gait aid is used and state the reason why the gait aid was only used partially. Continue with the rest of the survey (2) ____
- ○ No. Please explain why. Discontinue the survey. Thank the participant for their time. (3) ____

The rest of this survey are only for participants who have continued to use or partially using the recommended gait aid after the training program

Physical assessment: 4 metre walk test

Perform the 4 metre walk test (at a comfortable walking speed) **with the recommended gait aid.** Allow a practice trial before the test.

- [ ] Time required (in seconds, to one decimal place) (1)

____

- [ ] Gait speed (metre per second i.e. 4 divided by the time taken) (2)

____

- [ ] No. of steps (3) ____

- [ ] Step length (i.e. 4 m divided by the no. of steps) (4)

____

Physical assessment: Timed Up and Go (TUG) Test

Perform the TUG test (at a comfortable speed) **with the recommended gait aid.** Allow a practice trial before the test.

Ask the person with dementia or cognitive impairment to stand up from a standard height kitchen chair (46cm), walk three metres at their comfortable speed, turn, and return to the chair and sit down.

- [ ] Time required (in seconds, to one decimal place) (1)

____

Physical assessment: Figure of 8 walkway

Perform the Figure of 8 walkway test (at a comfortable speed) **with the recommended gait aid.** Allow a practice trial before the test.

Instructions to the person with dementia or cognitive impairment:

- Stand between the 2 cones (placed 5 feet apart i.e. 1.5 m), facing in one direction (the starting/stopping point is not marked "… in order to avoid influencing the movement planning for the task.")
- Begin walking at your <u>usual pace</u> when ready (the person chooses which direction to go around the cones)
- Stop when you return to the start position.

Instructions to the therapist:

- Timing: begin timing with the first step, and stop timing when the person with dementia assumes a side-by-side stance of the feet back at the start position.
- Accuracy: "The 2-ft (0.6 m) surround test boundary was not marked on the course. The tester determined the 2-ft (0.6 m) boundary area for the test setup and the relationships of the boundary to the testing space (e.g. distance from the hallway walls, floor markings, or landmarks) prior to testing, and estimated whether the test was completed within the boundary by comparison with the tester's mental map of the testing space."

☐ Time required (in seconds, to one decimal place) (1)

____

☐ Accuracy: does the person stay within the unmarked 2-foot (0.6 m) surround boundary of the cones? Write Yes or No response only. (2)

____

Repeat the Figure of 8 walkway test (at a comfortable speed) **with the recommended gait aid AND with a dual cognitive task.** Allow a practice trial before the test.

Instructions to the person with dementia or **cognitive** impairment:

- Stand between the 2 cones (placed 5 feet apart i.e. 1.5 m), facing in one direction (the starting/stopping point is not marked "… in order to avoid influencing the movement planning for the task.")
- Begin walking at your <u>usual pace</u> when ready (the person chooses which direction to go around the cones) and count backwards by 1's from 50 whilst walking.
- Stop when you return to the start position.

Instructions to the therapist:

- Timing: begin timing with the first step, and stop timing when the person with dementia assumes a side-by-side stance of the feet back at the start position.
- Accuracy: "The 2-ft (0.6 m) surround test boundary was not marked on the course. The tester determined the 2-ft (0.6 m) boundary area for the test setup and the relationships of the boundary to the testing space (e.g. distance from the hallway walls, floor

markings, or landmarks) prior to testing, and estimated whether the test was completed within the boundary by comparison with the tester’s mental map of the testing space.”

☐ Time required (in seconds, to one decimal place) (1)
____

☐ Accuracy: does the person stay within the unmarked 2-foot (0.6 m) surround boundary of the cones? Write Yes or No response only. (2)
____

☐ Counting errors: Write 1=Only 0 or 1 are incorrect responses, 2=Only 2 or 3 are incorrect responses, 3=Only 2 or 3 are correct responses, 4=Only 0 or 1 are correct responses, 5=Needs to stop (unable to walk and count at the same time). (3)
____

Ask the **participant** with dementia or cognitive impairment to rate the following statements:

	Strongly agree (1)	Somewhat agree (2)	Neither agree nor disagree (3)	Somewhat disagree (4)	Strongly disagree (5)
Walking with (state the type of gait aid) has improved my walking (1)	○	○	○	○	○
Walking with (state the type of gait aid) has made me feel safe (2)	○	○	○	○	○
Walking with (state the type of gait aid) feels right for me (3)	○	○	○	○	○

Ask the **participant with dementia or cognitive impairment** to provide comments for the above responses. Record the comments here.

____

Ask the **informal caregiver** to rate the following statements:

	Strongly agree (1)	Somewhat agree (2)	Neither agree nor disagree (3)	Somewhat disagree (4)	Strongly disagree (5)
The gait aid has improved the participant's steadiness in walking (1)	○	○	○	○	○
The gait aid has improved the participant's safety in walking (2)	○	○	○	○	○
Using the gait aid is appropriate for the participant (3)	○	○	○	○	○

Ask the **informal caregiver** to provide comments for the above responses. Record the comments here.

____

Give **your opinion (i.e. physiotherapist)** to rate the following statements:

	Strongly agree (1)	Somewhat agree (2)	Neither agree nor disagree (3)	Somewhat disagree (4)	Strongly disagree (5)
The gait aid has improved the participant's steadiness in walking (1)	○	○	○	○	○
The gait aid has improved the participant's safety in walking (2)	○	○	○	○	○
Using the gait aid is appropriate for the participant (3)	○	○	○	○	○

Give your (i.e. physiotherapist) comments for the above responses. Record the comments here.

____

Do you think the person with dementia/cognitive impairment has successfully retained the skill of safe and correct use of gait aid?

○ Yes (1)

○ Partially. Please state where their skill deficits are present (2) ____

○ No (3)

Discuss the follow-up findings with the person with dementia or cognitive impairment and their caregiver. What recommendations have you provided to them at the end of this follow-up session?

○ Recommend to continue using the gait aid in the long term at all times when up and ambulating (1)

○ Recommend to continue using the gait aid in the long term but only at some of the time, please state the situations when the person is recommended to use the gait aid. (2) ____

○ Do not recommend to continue using the gait aid. Please explain why. (3) ____

○ Other. Please state (4) ____

Do you (physiotherapist) have any further comments regarding the gait aid training program?

____

Please provide your (physiotherapist) name so that the research team can contact you in case of queries.

____

Thank you for completing the data collection survey. Please move to the next page to submit the survey online.

End of Block: Interview the person with dementia and the caregiver
