# Supplementary material for: The Potential for Effect of a Six-Week Training Program for Gait Aid Use in Older People with Dementia with Unsteadiness of Gait: A Pilot Study
Source: J Clin Med. 2023 Feb 16;12(4):1574. doi: 10.3390/jcm12041574 (PMC9967216; doi:10.3390/jcm12041574)
Supplement: Supplementary file 1 [file jcm-12-01574-s001.zip › Supplementary Material S6. Physio manual and protocol.pdf]

SUPPLEMENTARY MATERIAL S6: **TRAINING MANUAL FOR PHYSIOTHERAPISTS**

PROJECT: EFFECTIVENESS OF A SUPPORTED GAIT AID TRAINING PROGRAM FOR PEOPLE WITH DEMENTIA, AND FACTORS ASSOCIATED WITH SUCCESSFUL OUTCOMES

Authors:
Dr Angel Lee
Professor Keith Hill
Dr Elissa Burton
Dr Claudia Meyer
Associate Professor Susan Hunter

Contents

Introduction 3
Outline of the home-based gait aid training program 4
Outline of the physiotherapist education sessions 5
Objectives of the physiotherapist education sessions 5
Components of physiotherapist education sessions 5
Preparation for the education sessions 5
Education sessions 5
The educators 6
Study protocol 8
Background 8
Rationale 9
Research aims 9
Research hypothesis 9
Expected outcome 10
Ethics approval 10
Study design 10
Methodological approach 10
Setting and Participants 10
Inclusion criteria 10
Exclusion criteria 10
Participant recruitment strategy 11
Procedures 12
Approaches to provision of information to participants 12
Consenting procedure 12
Additional consenting procedure for WA participants 12
Baseline assessment (1st home visit) 13
Intervention 14
Follow-up assessments (2nd and 3rd home visits) 15
Final assessment (4th home visit) 15
Follow-up assessment (6 weeks after end of training program) 16
Data recording procedure 16
Risk management procedure 18
A guide to memory and communication strategies that can be used for people with dementia 19
Practical tips 19
A guide to strategies for decision-making in gait aid use and home practice 21

Systematic review 24
Background 24
Methods 24
Results 24
Conclusion 24
References 25
Appendix A 26
Cognitive Capacity Checklist 26
Appendix B 28
Appendix C 51
Rowland Universal Dementia Assessment Scale (RUDAS) 51
Appendix D 54
Appendix E 57
4 metre walk test 57
Appendix F 58
Time Up and Go test 58
Appendix G 59
Figure of 8 walkway 59
Figure of 8 walkway and dual cognitive task 60
Appendix H 61
Appendix I 71
Appendix J 81
Appendix K 100

Introduction

This training manual is developed for physiotherapists involved with implementing an extensive home-based gait aid training program for people with dementia and their carers.

The project "Effectiveness of a supported gait aid training program for people with dementia, and factors associated with successful outcomes" is funded by the Dementia Australia Research Foundation. The chief investigator is Dr Angel Lee. The co-investigators are Professor Keith Hill, Dr Elissa Burton and Dr Claudia Meyer. The associate investigators are Dr Fiona Connelly, Associate Professor Susan Hunter, Assistant Professor Plaiwan Suttanon, Professor Julie Stout and Professor Helen Dawes.

This manual provides an outline of the home-based gait aid training program and physiotherapist education sessions. It consists of a study protocol, procedures, data recording and risk management procedures that are related to the gait aid training program. It also includes a guide to memory and communication strategies that can be used for people with dementia, and strategies for decision-making in gait aid use and home practice.

The physiotherapist education sessions will take place before the commencement of the home-based training program. This manual is intended to be used in conjunction with the physiotherapist education sessions.

Outline of the home-based gait aid training program

The gait aid training program is a 6-week (comprising 4 home visits – see Figure S1 on page 7) program, with a further follow-up home visit 6 weeks after program completion (only for participants who were recommended by their physiotherapist to continue using the gait aid at the end of the training program).

Physiotherapists will be required to:

- Obtain consent from the person with dementia and their carer
- Conduct baseline assessments
- Work with the person with dementia and their carer to decide on the most suitable gait aid, based on assessment outcomes and clinical judgement, combined with environment scan and needs/preferences of the person with dementia and their carer
- Provide advice and an opportunity to practice with the selected gait aid during each of the four home visits. This will incorporate strategies to facilitate learning, and provision of advice for the carer to support home practice between home visits as deemed appropriate by the Physiotherapist
- Determine the suitability of gait aid practice by the person with dementia to be supervised by their carer between home visits
- Conduct assessments at the completion of each session, at the concluding 6-week session and at 12-weeks (if amenable to the person with dementia and their carer) to evaluate sustained learning of safe gait aid use

Outline of the physiotherapist education sessions

Objectives of the physiotherapist education sessions

- To explain the study protocol and methodology of the research
- To upskill in all assessment procedures
- To understand common strategies to facilitate learning of gait aid use by people with dementia, and how best to communicate these strategies to people with dementia and their carers
- To use scenarios as a mechanism to facilitate
  - gait aid decision making for people with dementia
  - decision making related to gait aid practice (supervised by their carer) between home visits
- To support the study physiotherapists in making decisions about gait aid use and practice for difficult cases
- To teach data recording and management procedures required for the project

Components of physiotherapist education sessions

Preparation for the education sessions

Physiotherapists will read the study protocol (pages 8-11), procedures (pages 12-17), and systematic review (pages 24) on gait aid training for older people (including people with dementia).

Education sessions

There will be two education sessions. The study Physiotherapists in Victoria and WA will undertake the first education session as a group via online meetings. The second session will be conducted in person as a practical session for assessment procedures (separately in WA and Victoria). It is anticipated there will be two study Physiotherapists in each of Perth and Melbourne.

The first session (online education of approximately 4 hours) includes:

- Review of Cognitive Capacity Checklist
- Consenting process for WA and Victoria
- Review of study protocol and methods
- Review of algorithm for gait aid use
- Review of all assessment procedures
- Overview of memory and learning strategies, and communication strategies for people with dementia
- Case study scenarios to be a basis for discussion to share ideas about challenges and solutions that may arise during the training program
- Strategies and support to guide decision-making in gait aid use and practice for people experiencing difficulties with the program and gait aid use
- Data recording and management procedures

The second session (face-to-face workshop of approximately two hours) includes:

- Practice of all assessment procedures for the home visits
- It is anticipated that some of the assessment procedures will be familiar to Physiotherapists with gerontological or neurological clinical experience.

The educators

All education will be conducted by experienced researchers and Physiotherapists / Exercise Scientists (Dr Angel Lee, Dr Elissa Burton, Professor Keith Hill, Dr Claudia Meyer) and, for the memory and learning strategy component, Associate Professor Susan Hunter (a physiotherapy lecturer and researcher with strong expertise in the area of motor skill learning in people with cognitive impairment, University of Western Ontario, Canada).

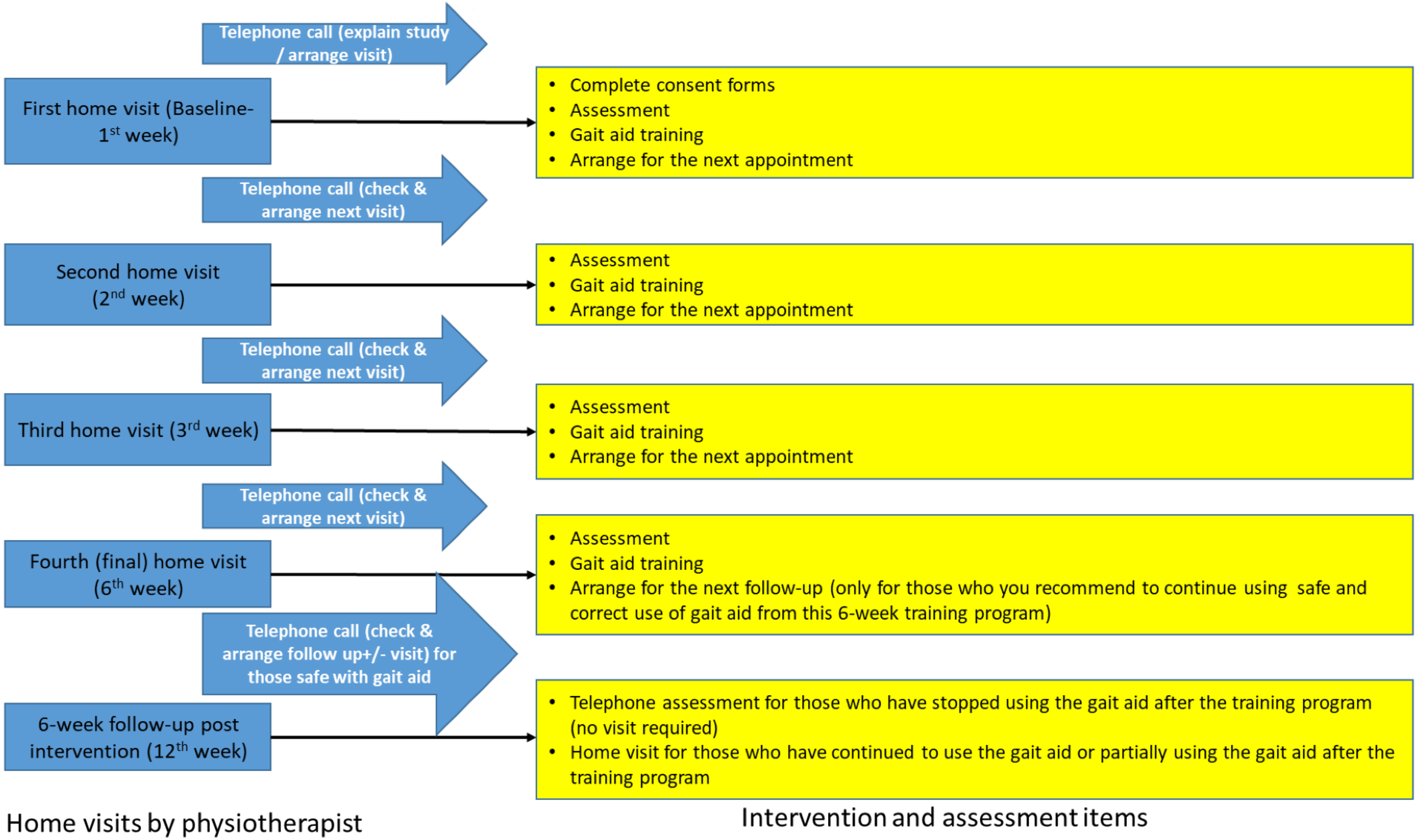

Figure S1. Intervention and assessment flow chart.

Study protocol

Background

Falls among older people have significant cost to the individual, their families / informal carers, and to the health and care systems. There is strong evidence for a range of single and multifactorial interventions to reduce falls in older people living in the community, [1, 2] however; most of this research has excluded people with dementia. Interventions such as exercise programs can improve balance and mobility and reduce falls risk in older people [2] and there is some emerging evidence of this for people with dementia.[3, 4] Yet those undertaking these interventions may still have some residual level of balance impairment or unsteadiness in walking, either related to their cognitive impairment, or to other health problems that are common among older people with dementia (e.g. stroke, osteoarthritis and neuropathy). Another common approach to improve steadiness of walking for older people generally is to provide them with a gait aid such as a stick or a walking-frame. Research indicates that appropriate use of a gait aid can improve walking stability for people with balance impairment.[5] There are additional benefits to using a gait aid, for example, it allows greater ability for ambulation and social participation.[6] Yet some research indicates that gait aid use may be independently associated with increased fall risk in people with dementia.[7]

Current approaches and important considerations for gait aid prescription. People with balance or gait impairments can access a gait aid through: (1) assessment by a health professional (e.g. physiotherapist or occupational therapist, through hospital or community setting); or (2) acquiring a gait aid through other means (e.g. purchase through pharmacist, loaned from another family member). If the gait aid is organised through a health professional, the practitioner should consider the severity of balance impairment, the acuity or chronicity of the cause of the balance impairment, and likelihood of improvement through medical (e.g. medication) or physical (e.g. exercise) approaches. If these considerations indicate the need for a gait aid, then further decision making is needed as to the level of support required for gait stability. The aim is to provide a gait aid that delivers the level of support required by the individual. Once the decision about type of gait aid has been determined, the gait aid needs to be adjusted to suit the individual's height.[8] Principles of which hand to use for a unilateral gait aid (e.g. a stick) and sequencing of the gait aid and leg movement are important to maximise stability, spread weight/load and optimise gait function. If instruction and training is not provided (e.g. gait aid purchased from a pharmacist), it may predispose the gait aid user to a fall. Education and practice with the gait aid can facilitate motor learning leading to the safe performance of gait.[9]

Gait aid use – benefit or risk? The contradiction in the research on gait aids conferring a benefit to stability versus causing increased falls risk needs further investigation. Previous research demonstrating improvements in stability with a gait aid has been limited to cognitively intact older people. We do not know if use of a gait aid is beneficial for older people with dementia. Factors that could link gait aid use to elevated fall risk in people with dementia include underlying neurodegenerative processes, incomplete motor learning, limited insight into safe gait aid use and increased cognitive demands related to attentional processing and neuromotor control.[10] Whilst neurodegenerative processes are not yet modifiable, motor skills and the ability to adapt walking patterns using a gait aid to move around one's home and community can potentially be learnt by people with dementia.[11] A systematic review on motor-skill learning in people with Alzheimer's Disease (a type of dementia) demonstrated intact implicit motor-learning capabilities across all studies; importantly, this occurred even in the presence of severe explicit memory impairment.[12]

Anecdotally, we are aware that health professionals can exercise too much or too little caution when providing gait aids to people with dementia. While some omit consideration of the disease-specific changes of people with dementia, others go to the opposite extreme of withholding access to a gait aid because of the perceived risk of its inappropriate use. Thus, there is a need to understand and guide the decision making process among health professionals. This research aims to fill the evidence gap to support health professionals in making decisions regarding gait aid use for older people with dementia who have balance impairment. It also aims to inform the refinement of a decision support tool (algorithm) that was developed in a previous study. With the use of a home-based gait aid-training program, it will test and support the practice in gait aid use and gait aid recommendations for this population who has a high risk of falls.

Rationale

To improve practice in gait aid use and gait aid recommendations for older people with dementia through a home-based gait-aid training program, ultimately aiming to reduce falls risk, and improve safety for people with dementia.

Research aims

a) To assess the feasibility and safety of a home-based gait aid-training program for older people living with dementia who have balance impairment.

b) To assess the ability of people with dementia who are unsteady to safely and correctly use a physiotherapist recommended gait aid within an initial training session; and whether a 6-week gait aid training program supplemented by home practice results in further improvements for the person with dementia to acquire and maintain the skill of safe gait aid use.

c) To test the accuracy of a decision support tool (algorithm developed by an expert panel* from a previous study) to aid decision making about whether or not a person with dementia will be likely to be able to learn to safely use a walking aid.

**Expert panel of experienced falls prevention/gait/mobility researchers, experienced practitioners working with people with dementia in hospital and community settings, a representative from policy and planning, representatives of stakeholder community care organisations (representing older people, people with dementia, and informal carers), and informal carers of older people with dementia.*

Research hypothesis

a) It is feasible and safe to conduct a home-based gait aid-training program for older people living with dementia who have balance impairment.

b) A small proportion of people with dementia (e.g. those with a recent diagnosis of dementia or those with mild dementia) who have balance impairment will be able to learn to safely and correctly use a gait aid **<u>within a single training session</u>** when the gait aid is provided.

c) A 6-week gait aid training program supplemented by home practice will be sufficient for some additional people with dementia to acquire and maintain the skill of safe gait aid use.

d) The algorithm* is accurate in classifying an older person with dementia who has balance impairment to be able to use a gait aid safely and effectively with a home-based gait aid-training program.

*Please note: The algorithm is **not** intended to guide physiotherapists in choosing the right type of gait aid for the person with dementia but rather to provide guidance (if tested to be helpful) **if any**

gait aid can be safely used by people with dementia who have balance impairment. The decision of which type of gait aid to use will be decided by the physiotherapists taking into account the level of support required (based on assessment and factors such as level of unsteadiness, unilateral pain), the common environmental constraints to be accessed by the person with dementia, and preference of the person with dementia and their informal carer. The algorithm may be further refined by the Expert Panel after the results of the pilot trial are reviewed.

Expected outcome

Results regarding the feasibility, safety and perception of safety and appropriateness of gait aid use (from older people with dementia, informal carer and the physiotherapist) will be generated from the six-week gait aid-training program. Other results regarding gait performance measures, adherence to gait aid use and home practice will also be obtained.

This home-based gait aid-training program will be feasible, safe, and meets the expectations and preferences of older people with dementia, their informal carers and physiotherapists.

Ethics approval

Ethics was approved by Monash University Human Research Ethics Committee (Project ID 25104), reciprocal approval from Curtin University Human Research Ethics Committee (Project ID:HRE2020-0614) and Bolton Clarke Human Research Ethics Committee (Project ID 223).

Study design

Methodological approach

This feasibility study will evaluate a home-based gait aid-training program designed for older people with dementia who have balance impairments. The results from this study will be used to test the accuracy of an algorithm (developed in another study, Monash University HREC approval project ID: 23238, Curtin University HREC approval project ID: HRE2020-0118 and Bolton Clarke HREC approval project ID: 216) in correctly classifying people with dementia who have balance impairment that are likely to be able to safely utilise a gait aid with training.

Setting and Participants

Older people (n=40) formally diagnosed with dementia, those having a significant cognitive impairment (recorded in the case notes of participating community care organisation) or those receiving dementia supplement in their home care packages, and their informal carers will be recruited from Victoria and WA.

Inclusion criteria

- Older people aged ≥65 years formally diagnosed with dementia, or those having a cognitive score (recorded in case notes from community care organisation, if recruited through this avenue) of ≤22 on the Rowland Universal Dementia Assessment Scale (RUDAS),[13] or ≤ 23 on the Mini-Mental State Examination,[14] or those who receive a dementia supplement in their home care packages.
- Have had at least one fall in the preceding 12 months, or report unsteadiness when walking or turning (including report by informal carer), and
- Willing to participate in a 6-week gait aid use training program, and
- Informal carer's availability to support and reinforce correct gait aid use between physiotherapist home visits (if applicable).

Exclusion criteria

- The person with dementia is already using a gait aid, or

- The person with dementia refuses to use a gait aid, or
- The person with dementia is assessed as requiring manual assistance for walking with or without a gait aid or that the person is normally not able to walk e.g. bed or wheelchair bound people, or
- The person with dementia or the informal carer plans to be away from home for a substantial period during the 6-week training program

Participant recruitment strategy

A number of community care organisations in Victoria (Bolton Clarke) and Western Australia (Alzheimer's WA, Cockburn Care, Amana Living, and Brightwater) will facilitate recruitment of people with dementia/cognitive impairment and their informal carers.

If the target number of 40 older people with dementia/cognitive impairment and their informal carer cannot be reached with these participating organisations, participants will also be recruited from the general community through promotion of research to organisations such as Dementia Australia, Council of the Ageing, Carers' WA, Carers' VIC, and the Rehabilitation Ageing and Independent Living (RAIL) research centre (connections with the community of Mornington Peninsula region in Victoria).

In addition, informal carers who provided their contact number in a previous study indicating their willingness to participate in this training program will also be contacted to determine their interest in being involved.

Procedures

Approaches to provision of information to participants

Participation in the training program is voluntary. People with dementia/cognitive impairment and their informal carers who are interested in participating in the study can provide their names and contact details to the research team.

The physiotherapists will contact the people with dementia/cognitive impairment and their informal carers by phone to: 1) explain participation, 2) ascertain they understand the nature of the study and 3) screen for inclusion/exclusion criteria. A written Participant Information and Consent Form (containing the study information and details about the first session) will be sent to the person with dementia/cognitive impairment and their informal carer prior to the first home visit.

Consenting procedure

The physiotherapist will arrange for a home visit with the person with dementia/cognitive impairment and their informal carer. The physiotherapist will administer the Cognitive Capacity Checklist for the person with dementia/cognitive impairment to screen for their capacity to consent (Appendix A). This checklist includes asking the person with dementia/cognitive impairment to clarify, in their own words, what involvement will entail (rather than only being asked to respond yes or no to the questions).

Written consent from the person with dementia/cognitive impairment (those with capacity to consent as indicated by the checklist) and informal carer will be gained at the start of this initial home visit. The informal carer of the person with dementia/cognitive impairment will provide consent for their own role in the project.

For people with dementia/cognitive impairment who are deemed <u>not having the capacity to consent</u> using the Cognitive Capacity checklist, the physiotherapist will ascertain if the person has either or both of the following:

- Advanced care directive (the physiotherapist will check that the research does not contravene this)
- A medical treatment decision maker

Informed consent will be sought from this medical treatment decision maker if there is an appointed medical treatment decision maker. If not, their informal carer will provide informed consent on behalf of the person.[15] The informal carer is the first of the following with a close and continuing relationship with the person:

- The spouse or domestic partner
- The primary carer of the person
- The oldest adult child of the person

Additional consenting procedure for WA participants

For people with dementia/cognitive impairment who are <u>not able to provide consent</u>, the physiotherapist will request the *research decision maker* (i.e. in this case their carer - who will make the decision on behalf of the person) to take the GAA Medical Research Decision Form to their GP (independent medical practitioner) for signing. A short letter will be provided by the research team to their GP explaining the study and the reason for the GP to complete and sign the GAA form.

Once the GAA form is signed, the physiotherapist will complete the researcher actions section of the form. The physiotherapist will then contact the Joint Lead Investigator, Dr Fiona Connelly (Armadale Health Service) and discuss the accuracy and compliance to the GAA act and Dr Connelly will then sign the form. The WA site manager (EB) will send a GAA Medical Research Decision Report to the WA Department of Health within 15 days of each recruitment of a person with dementia/cognitive impairment who is not capable of providing consent.

Separate Participant Information and Consent Forms will be used for (1) people with dementia/ cognitive impairment (for those with capacity to consent), (2) informal carers of the people with dementia/cognitive impairment, and (3) informal carer (research decision maker) who is consenting for the person with dementia/cognitive impairment if the person does not have the capacity to consent. The physiotherapist will obtain written consent from all relevant consenting parties before the training begins.

There will be an opportunity to re-negotiate consent during the study. The participants can discuss it directly with the physiotherapist or contact the research team. They may withdraw from the study at any time.

Baseline assessment (1st home visit)

All assessment items of physical performance will be undertaken with the physiotherapist providing close supervision for the person with dementia/cognitive impairment, and steady them if needed (which is routine practice for physiotherapy assessments in older people).

Assessment will include (see Appendix B for data collection survey to be used):

- ***Demographics:*** including age, gender, type of dementia (e.g. Alzheimer's, Vascular, Lewy Body), years living with dementia, other medical history, living arrangement, current self-reported physical capacity, current exercise/physical activity participation and falls in the last 12 months. For this study, a fall will be defined as "inadvertently coming to rest on the ground, floor or other lower level, excluding intentional change in position to rest in furniture, wall or other objects."[16]
- ***Cognition:*** Rowland Universal Dementia Assessment Scale (RUDAS-see Appendix C)
- ***Algorithm:*** apply assessment items from the algorithm (Appendix D)
- ***Temporal and distance measures of gait:***
- 4 metre walk test[17] (at comfortable walking speed) with and without the recommended gait aid. Time, gait speed (metre per second), number of steps and step length data will be collected (See Appendix E).
- Timed Up and Go test–asking the participants to stand up from a standard height kitchen chair, walk three metres at their comfortable speed, turn, and return to the chair and sit down with and without the recommended gait aid. Time data will be collected (see Appendix F).
- Timing performance and accuracy walking a figure of 8 walkway, and repeating this, using a dual cognitive task (serial subtraction by 1 from 50) with and without the recommended gait aid. Time, number of steps and accuracy (does the person stay within the unmarked 2-foot i.e. 0.6 m surround boundary of the cones? yes or no response) (see Appendix G)
- ***Perception rating of safety and appropriateness of gait aid use:*** The physiotherapist, the person with dementia/cognitive impairment, and the informal carer will each rate their perception of the safety and appropriate use of the gait aid on a Likert scale, and provide written comments to support the rating.

The outcome measures collected at the 1st home visit will be:

- Temporal and distance measures of gait
- Perception rating of safety and appropriateness of gait aid use

A falls diary and a home practice diary will be given at the initial visit to the participants. The physiotherapist will instruct the participants how to fill them out.

Intervention

All consenting participants (except those who are deemed unsafe by the physiotherapist based on their clinical judgement) will participate in the gait aid-training program.

Combining the assessment items of the algorithm, and the additional physical performance and cognition assessments, together with a discussion with the participant and their informal carer about preferences, the physiotherapist will determine the most appropriate gait aid to trial for the six-week training program. The physiotherapist will provide practice with the selected gait aid in this and subsequent home visits, with consistent verbal and other cueing to reinforce safe and correct use.

The program will consist of:

- A discussion by the physiotherapist with the participant and their carer about the results of the assessments, their judgement on a suitable walking aid to use, and the preferences of the person with dementia/cognitive impairment and their carer;
- Determining the most appropriate gait aid to start the training trial with;
- Practice and instruction with use of the selected gait aid during the home visit;
- Discussion and training of the carer with respect to supervision, prompting approaches, safety with the gait aid for the person with dementia/cognitive impairment;
- Determining whether or not the person with dementia/cognitive impairment will be safe to practice walking with the gait aid with their informal carer between the home visit sessions and in these cases, provision of written prompts and support information for the person with dementia/cognitive impairment and their carer. If the person with dementia/cognitive impairment is deemed not safe to use the gait aid without the physiotherapist present, the gait aid will not be left with them. This status will be reviewed at each home visit. This means that the person with dementia/cognitive impairment will only practise using the gait aid with the physiotherapist at the home visits, and these participants will still be included in the study.

The Physiotherapist will provide up to four home visits over the six week intervention (baseline=1st home visit at week 1, 2nd home visit at week 2, 3rd home visit at week 3, and 4th home visit at week 6). At each home visit (approximately two hours for week 1 and 6, and one hour for week 2 and 3), the physiotherapist will:

- Provide practice with the selected gait aid, with consistent verbal and other cueing to reinforce safe and correct use;
- Provide memory aids and other prompts to the participants, to support regular and appropriate use of the gait aid (e.g. correct hand if a unilateral gait aid, correct sequence of use). In addition, an information sheet will be provided to the informal carer to outline strategies to reinforce safe and correct gait aid use between visits by the physiotherapist, if the physiotherapist decides this is safe;
- Reassess the participant and carer's safety in undertaking home practice with the gait aid between the physiotherapist home visits. In some instances this may require changing the

type of gait aid being used part way through the program (e.g. if it becomes apparent the participant is not having success with using a cane, they may subsequently be trialled with a walking frame);

- Provide instructions for the participant and carer about the nature of home practice with the gait aid between physiotherapist home visits, if this is decided to be safe; and
- Document the nature/content of the practice session, memory aids used, and instruction for home practice (if used), so that detail of the intervention home visits can be collated and reported.

Follow-up assessments (2nd and 3rd home visits)

Assessment will include (see data collection surveys to be used- Appendix H for 2nd home visit and Appendix I for 3rd home visit):

- ***Perception rating of safety and appropriateness of gait aid use:*** The physiotherapist, the person with dementia/cognitive impairment, and the informal carer will each rate their perception of the safety and appropriate use of the gait aid on a Likert scale, and provide written comments to support the rating.
- ***Falls***. Number of self-reported falls since last home visit (using a falls diary provided by the physiotherapist), details of falls and whether the gait aid was being used or was thought to have contributed to the fall.
- ***Adherence.*** Number of home practice sessions carried out by the person with dementia/cognitive impairment compared to instructions of frequency of practice by the physiotherapist if applicable (using a practice diary provided by the physiotherapist).
- ***Adverse events.*** Adverse event that occurred with the home practice e.g. falls, injury, pain

The outcome measures collected at the 2nd and 3rd home visits will be:

- Perception rating of safety and appropriateness of gait aid use
- Falls in the last week, and details associated with these
- Adherence to home practice sessions
- Adverse events associated with home practice

Final assessment (4th home visit)

In addition to the follow-up assessments at 2nd and 3rd home visits, the following assessments will be made (see Appendix J or data collection survey to be used):

Temporal and distance measures of gait:

- 4 metre walk test (at comfortable walking speed) with and without the recommended gait aid. Time, gait speed (metre per second), number of steps and step length data will be collected.
- Timed Up and Go test– asking the participants to stand up from a standard height kitchen chair, walk three metres at their comfortable speed, turn, and return to the chair and sit down with the recommended gait aid. Time data will be collected.
- Timing performance and accuracy walking a figure of 8 walkway, and repeating this, using a dual cognitive task (serial subtraction by 1 from 50) with and without the recommended gait aid. Time, number of steps and accuracy (does the person stay within the unmarked 2-foot i.e. 0.6m surround boundary of the cones? yes or no response)

Physiotherapist judgement (for each person with dementia) on:

- Adequacy of the length of the training program;
- Adequacy of the frequency of home visits;
- Any changes they would recommend to the standard intervention program, based on the responses of the person with dementia/cognitive impairment.

The outcome measures collected at the final home visit will be:

- Temporal and distance measures of gait
- Perception rating of safety and appropriateness of gait aid use
- Falls in the last two weeks
- Adherence to home practice sessions
- Adverse events associated with home practice

At this final visit, a collaborative decision (between the physiotherapist, person with dementia/cognitive impairment and their carer) will be made as to whether the training program has been beneficial, taking into account the changes in gait and mobility data over the last six weeks and whether ongoing use of the gait aid will occur. If so, the participants will be given that gait aid to use long term (i.e. after the study has been completed).

A flow chart for the home visits, intervention and assessment items is presented in Figure S1 on page 7.

Follow-up assessment (6 weeks after end of training program)

A follow-up will be conducted at 6 weeks after the end of the training program but **only** be for those participants who were considered suitable to continue using a gait aid at the end of the six week training program.

At this follow-up, the Physiotherapist will call the participants to ask if they have continued to use the gait aid, partially using the gait aid or have stopped using the gait aid after the training program.

If the person has stopped using the gait aid after the training program, the physiotherapist will complete a short assessment on the phone only (see Appendix K for data collection—top half of the survey). No home visit is required.

If the person has continued to use or partially using the gait aid, the physiotherapist will arrange a home visit to review (see Appendix K for data collection survey to be used):

- If the person with dementia has continued to use the recommended gait aid (and whether this is at all times, or for certain tasks, and % of the time walking that the gait aid is being used).
- Whether there have been any adverse events or falls since the end of the training program and whether the gait aid was being used at the time, and details about the fall / adverse event. The participants will continue to use the falls diary in the 6 weeks after the training program (but not for those that were not included in this follow-up).
- Repeat the assessments of gait aid performance used at the final training assessment.

The Physiotherapist will discuss the follow-up findings in the context of continuing use of the gait aid with the person with dementia and their carer in the longer term.

Data recording procedure

All data will be recorded by the physiotherapist conducting the home visits using data collection surveys. A survey for each home visit has been developed using the survey platform (Qualtrics.com) that includes the relevant assessment items for each visit (see Appendix B, H, I, J, K). There will be

five unique URL survey links to the home visits, i.e. a link for the 1st home visit, one for the 2nd home visit, one for the 3rd home visit, one for the 4th home visit and one for the 6 weeks post-program follow-up.

All data must be entered online (either at the home visit or subsequently if you choose to use the paper-based version of the survey at the home visit) using the unique URL link provided for each survey. Please make sure you use the correct URL survey link for the home visit that you are conducting.

The survey data for the four (and post-program) home visits will be downloaded at project completion for analysis.

Risk management procedure

There is a risk of falls during the gait aid-training program for the person with dementia/cognitive impairment. However, this will be minimised through close supervision by the physiotherapist during the home visits (to steady the person with dementia/cognitive impairment if they appear unsteady during the practice). If the physiotherapist considers it safe to leave the gait aid for practice between the home visits, this will be provided with instructions regarding the level of supervision by the carer and acceptance by the carer that they will be able to provide this level of supervision. If the participant is at a major risk of falls (according to physiotherapist's clinical judgement) from learning to use the recommended gait aid, the physiotherapist will discontinue the trial and recommend a consultation with their medical practitioner to review the participant's falls risk and management.

Steps to follow if the person with dementia/cognitive impairment falls during the home visit. The physiotherapist will:

- Check the person for injury and apply first aid if required.
- Help the person seek medical advice if required through his/her medical practitioner. If the injury is serious, the physiotherapist will call for an ambulance and stay with the person until help arrives.
- The physiotherapist will report the incident to the research team. The research team will work together with the physiotherapist, the person with dementia/cognitive impairment, their informal carer and their medical practitioner (if applicable) to decide whether ongoing participation in this study is appropriate.
- Report the incident to the community care agency if the person is receiving services from a community care agency. Internal policies and procedures will need to be followed.

Steps to follow if the person with dementia/cognitive impairment falls during home practice of gait aid in between the home visits, the informal carer will:

- Seek medical help for the person with dementia/cognitive impairment through his/her medical practitioner or call for an ambulance if needed.
- Report the incident to the research team. The research team will work together with the physiotherapist, the person with dementia/cognitive impairment, their informal carer and their medical practitioner (if applicable) to decide whether ongoing participation in this study is appropriate.
- Discontinue the home practice until the next physiotherapy home visit when the physiotherapist will review the home practice recommendations.
- Report the incident to the community care agency if the person is receiving services from a community care agency. Internal policies and procedures will need to be followed.

In either situation if a serious injury occurs, the research team will report the serious adverse event to the Ethics committee.

Use of a gait aid is a common intervention for older people who are unsteady in their mobility. In light of the high risk of falls for people with dementia, this study aims to support an adapted approach to maximise safety of gait aid use which may ultimately reduce falls for people with dementia.

For the safety of physiotherapists doing home visits, please inform your research site manager when you are at the participant's home and when you finish your home visit and arrive back home safely.

A guide to memory and communication strategies that can be used for people with dementia

A systematic review of motor-skill learning in people with Alzheimer's disease demonstrated intact implicit motor-learning capabilities across all studies.[12] Implicit motor learning refers to the recurrent practice of a task until the learner reaches a stage where he/she does not require to develop conscious rules to guide performance.

Importantly, this implicit learning can occur even in the presence of severe explicit memory impairment (before advanced stage of dementia). Explicit memory impairment is "the conscious recollection of facts, ideas and events combined with a decreased capacity for explicit learning and error detection limit the methods by which people with dementia can relearn mobility activities"[13] These will not be effective in people with dementia who have impaired short term memory.

To facilitate implicit motor learning in people with dementia, there are factors that need to be considered. In particular, there is a need for repeated and consistent practice conditions to learn a motor task (i.e., repeated running of the same motor task which does not require an intact episodic memory). People with dementia are likely to experience difficulty learning from a variable practice regimen because of the difficulty with switching tasks and dividing attention.[11]

Several factors can be addressed to create the best chance for acquisition, retention, and transfer of gait aid skills into everyday life for people with dementia. Key elements include repetitive hands-on training that relies of demonstration and imitation rather than verbal instruction, practice to establish both proper and consistent use of good gait aid technique. The demonstration and imitation approaches can build on motor learning skills in the person with dementia, which remain mostly intact in Alzheimer's disease and other dementias with more cortical, rather than subcortical brain tissue loss. Because of this approach relies on the motor system and behaviours that are associated with the gait aid as a familiar stimulus, the gait aid user may not need to have any explicit memory of the training or the trainer, but still be able to employ their gait aid use skills accurately and consistently.

"Errorless" learning is a learning paradigm based on the implicit learning principles of high repetition and low variability and has been used successfully to teach people with dementia in relearning tasks of activity of daily living e.g. eat independently, use a mobile phone.

The concepts of "errorless" learning are based on:

1) Practice conditions that are designed so that inaccurate performance during the learning process is prevented or minimised

2) Repetitive practice without errors during practice and this may consolidate memory of correct performance within the implicit memory system

3) Errorless learning over trial-and-error learning may be preferable in people with dementia

4) Elimination or reduction in errors in an errorless learning session may reduce frustration and increase participation of the person with dementia

Practical tips

- Base training on actions rather than words. Avoid verbal explanations or descriptions. Some key words can be developed as cue to support accurate and consistent use ('unlock wheels first')

- Provide praise and other positive feedback with words and gestures. It may be useful to use a visual chart of achievements (e.g. white board with ticks or something more meaningful)
- Training should be based on demonstration by the trainer, followed by rehearsal by the user to the point of consistent accurate performance.
- Training should commence in consistent, non-distracting conditions, and be repeated until the skill is established.
- Distribute training across multiple sessions to facilitate consolidation of skills
- Once the skill is established start to build the skill across different contexts such as
    - Different rooms of the home (e.g. bathroom, bedroom)
    - Different conditions (e.g. TV on, visitors present, while conversation is going on). If the person's level of skill becomes inaccurate, consider additional rehearsal to solidify the skills. If the skill level has deteriorated too much or cannot be established under different conditions, note that safe ambulation is to be supported through reducing distractions at critical times of gait aid use.
- Tailor training to the needs and ability of the person with dementia.
- Select training tasks that are functionally relevant
- Use frequent and consistent practice to limit variation and provide routine for the person
- Give consistent visual and / or verbal feedback (e.g. use of memory aids, reminders, visual and verbal cues)
- Use modelling (demonstrate the task/step to the person and ask the person to practise the task/step before additional tasks/steps are demonstrated and practised)
- Avoid practice under multi-tasking conditions (i.e. reduce cognitive loading during training) *Although the use of a gait aid itself might be considered a dual task, this refers to avoiding other concurrent tasks while walking with the gait aid.*
- Train in an environment that closely resembles the one in which the skill is going to be used
- Reduce fatigue in learning (i.e., use of distributed rather than mass practice during training)
- Limit the number of tasks so that each task can be practised multiple times in a session

A guide to strategies for decision-making in gait aid use and home practice

The aim is for all people with dementia/cognitive impairment recruited for the study to undergo the assessments, and then to progress to the trial, unless the physiotherapist deems (based on their clinical judgement) that it would not be safe to do so. In such cases, the trial will stop at that point (anticipated to be a small proportion of participants).

For those who require close supervision, but are deemed safe, they will still be able to progress to the training trial for the "supervised" gait aid training program component (home visits by physiotherapist), however, may not be involved in the "home" gait aid practice (with carer supervision) component.

Factors that may inform decision making regarding gait aid use and self practice at home include:

- Level of independence and steadiness
- Ability to adapt to safe gait aid use
- Home environment constraints (e.g. some homes may have insufficient space / access for a rollator in the toilet or bathroom; and / or a need to negotiate indoor or outdoor steps
- Amount of time their carer is likely to be able to spend supervising gait aid use and practice

Below are two scenarios for physiotherapists to discuss and share their thoughts on decision making regarding gait aid use for people with dementia and home practice of gait aid use (supervised by their carer) between home visits.

<u>Scenario 1:</u> Mr Bernie Smith, 85 year old man, lives at home with 72 year old wife (Margaret) who is generally well (mild right knee OA, hypertension; community ambulant with single point stick, no gait aid indoor).

Medical history (Bernie):

- Moderate severity mixed Alzheimer's disease /vascular dementia diagnosed 5 years ago
- Right hip replacement 8 years ago, some residual pain / weakness, antalgic gait
- Mild emphysema, shortness of breath after walking to the next corner in the street (approx 150m)

Mobility (Bernie):

- Still enjoys gardening, but doing less outdoors at home. Rarely walks away from home in past few months (he and his wife are worried about falls when walking away from home)
- Unsteady gait, especially on turning. Three falls in past 12 months, two indoors when turning, one in the garden (tripped over hose)

<u>Questions to consider (make notes and discuss as a group):</u>

1. What factors will influence your choice of a walking aid for Bernie?
2. What gait aid would you try initially for Bernie (single point stick or rollator)?
3. What specific activities would you trial with Bernie in the first session with the gait aid?
4. If you commence trialling Bernie with a single point stick, what will you be looking for to be confident that he could have the single point stick for use with Margaret's supervision for home practice after the first session?

5. If you commence trialling Bernie with a rollator, what will you be looking for to be confident that he could have the rollator for use with Margaret's supervision for home practice after the first session?

Physiotherapist notes:

____
____
____
____
____
____
____
____
____
____
____

Several days after the first home visit when you left the gait aid for supervised use by Bernie, Margaret rings you to say that Bernie has had several near falls during the home practice with her supervision. What do you ask Margaret? What do you recommend for Margaret / Bernie to do?

Physiotherapist notes:

____
____
____
____
____
____

<u>Scenario 2:</u> Mrs Glenys Parker, a 78 year old lady, lives at home alone. Her retired son (Jack) spends a number of hours each day visiting.

Medical history (Glenys):

- Mild severity Alzheimer's disease diagnosed 2 years ago
- Diabetes
- Bilateral cataracts, awaiting surgery
- Old right CVA, fairly good recovery, occasional right foot scuffing, good upper limb function
- Intermittent dizziness – no formal testing or treatment.

Mobility (Glenys):

- Mainly restricted to mobility at home (indoors and outdoors). Has a single step to go out to the back yard
- Moderate unsteadiness in walking and turning, tends to furniture walk to keep steady

- Two falls in the past year, both when home alone (indoors), cannot recall circumstances, but had bruising on knee and face.

Questions to consider (make notes and discuss as a group):

1. What factors will influence your choice of a walking aid for Glenys?
2. What gait aid would you try initially for Glenys (single point stick or rollator)?
3. What specific activities would you trial with Glenys in the first session with the gait aid?
4. If you commence trialling Glenys with a single point stick, what will you be looking for to be confident that she could have the single point stick for use with Jack's supervision for home practice after the first session?
5. If you commence trialling Glenys with a rollator, what will you be looking for to be confident that she could have the rollator for use with Jack's supervision for home practice after the first session?

Physiotherapist notes:

____
____
____
____
____
____
____
____
____
____

At your first follow up visit with Glenys, she appears to be becoming more confident and using the gait aid generally well.

1. What type of activities will you trial with Glenys on this visit to help your decision about whether or not she can progress to using the gait aid on her own (i.e. not limited to just when Jack is visiting and can supervise her).
2. What factors will you consider in deciding whether or not she can progress to using the gait aid all of the time (i.e. not limited to just when Jack is visiting and can supervise her).

Physiotherapist notes:

____
____
____
____
____

Systematic review

To date, there have been no reviews that have investigated the potential effects of gait aid prescription in older people with mobility problems or fall risks. The aim of this review was to integrate and describe the research evidence relating to the effectiveness of gait aid prescription for improving spatiotemporal gait parameters, balance and / or reducing falls, and how it affected the safety and adherence to gait aid use among these community-dwelling older people.

The abstract from the systematic review paper (accepted in press in Disability and Rehabilitation) is presented below.

Background

To integrate the evidence of gait aid prescription for improving spatiotemporal gait parameters, balance, safety, adherence to gait aid use and reducing falls in community-dwelling older people.

Methods

Seven health databases were searched to June 2021. Experimental studies investigating gait aid prescription (provision and instruction for use) for older people, reporting gait parameters, balance, falls, and safety of or adherence to gait aid use were included. Mean differences with 95% confidence intervals of gait and balance outcomes in participants at the program's last follow-up were analyzed. The safety of and adherence to gait aid use were described.

Results

Eight studies were included (N=555 older people). No meta-analyses could be performed. Five studies used a single gait aid instruction session. Gait aid prescription had inconsistent effects on gait velocity, and no reported benefits in reducing gait variability in older people with mobility problems or fall risks, including Parkinson's or Alzheimer's disease. No study investigated gait aid prescription on falls and balance performance. Effects on safety and adherence to gait aid use were unclear.

Conclusion

Research is needed to investigate the benefits of extensive gait aid training in older people with mobility problems, including those with dementia or high falls risk.

References

1. Gillespie, L. D.; Robertson, M. C.; Gillespie, W. J., et al. Interventions for preventing falls in older people living in the community, *Cochrane Database of Systematic Reviews*. **2012,** DOI 10.1002/14651858.CD007146.pub3.
2. Sherrington, C.; Tiedemann, A.; Fairhall, N. J., et al. Exercise for preventing falls in older people living in the community, *Cochrane Database of Systematic Reviews*. **2016,** DOI 10.1002/14651858.CD012424.
3. Suttanon, P.; Hill, K.; Said, C., et al. Feasibility, safety and preliminary evidence of the effectiveness of a home-based exercise programme for older people with Alzheimer's disease: a pilot randomized controlled trial, *Clinical Rehabilitation*. **2012,** ***27,*** 427-738.
4. Burton, E.; Cavalheri, V.; Adams, R., et al. Effectiveness of exercise programs to reduce falls in older people with dementia living in the community: a systematic review and meta-analysis, *Clinical interventions in aging*. **2015,** ***10,*** 421-434.
5. Härdi, I.; Bridenbaugh, S. A.; Gschwind, Y. J.; Kressig, R. W. The effect of three different types of walking aids on spatio-temporal gait parameters in community-dwelling older adults, *Aging Clinical and Experimental Research*. **2014,** ***26,*** 221-228.
6. Bateni, H.; Maki, B. E. Assistive devices for balance and mobility: Benefits, demands, and adverse consequences, *Archives of Physical Medicine and Rehabilitation*. **2005,** ***86,*** 134-145.
7. Fernando, E.; Fraser, M.; Hendriksen, J.; Kim, C. H.; Muir-Hunter, S. W. Risk Factors Associated with Falls in Older Adults with Dementia: A Systematic Review, *Physiotherapy Canada Physiotherapie Canada*. **2017,** ***69,*** 161-170.
8. Crosbie, J.; Armstrong, E.; Kempson, J. Is walking aid height critical?, *Australian Journal of Physiotherapy*. **1992,** ***38,*** 261-266.
9. Boraxbekk, C. J.; Hagkvist, F.; Lindner, P. Motor and mental training in older people: Transfer, interference, and associated functional neural responses, *Neuropsychologia*. **2016,** ***89,*** 371-377.
10. Hunter, S. W.; Divine, A.; Omana, H., et al. Effect of Learning to Use a Mobility Aid on Gait and Cognitive Demands in People with Mild to Moderate Alzheimer's Disease: Part I - Cane, *Journal of Alzheimer's disease : JAD*. **2019,** DOI 10.3233/jad-181169.
11. Dick, M. B.; Hsieh, S.; Bricker, J.; Dick-Muehlke, C. Facilitating acquisition and transfer of a continuous motor task in healthy older adults and patients with Alzheimer's disease, *Neuropsychology*. **2003,** ***17,*** 202-212.
12. Kuzis, G.; Sabe, L.; Tiberti, C.; Merello, M.; Leiguarda, R.; Starkstein, S. E. Explicit and implicit learning in patients with Alzheimer disease and Parkinson disease with dementia, *Neuropsychiatry Neuropsychol Behav Neurol*. **1999,** ***12,*** 265-269.
13. White L, Ford MP, Brown CJ, Peel C, Triebel KL. Facilitating the use of implicit memory and learning in the physical therapy management of individuals with Alzheimer disease: a case series. J Geriatr Phys Ther. 2014 Jan-Mar;37(1):35-44.

Appendix A

Cognitive Capacity Checklist

Can the person provide their own consent?

Please tick yes or no to each statement based on your knowledge of the person:

	YES	No
Can the person describe, in their own words, what the project is about after you have explained it to them?		
Would the person know that it is up to them only to decide whether they want to be in the project?		
Can the person describe, in their own words, what benefit they might have from being in the project?		
Can the person describe, in their own words, what the risks and inconvenience are from being involved in the project?		
Would the person understand that they can complain about the project to any one of the following people: a member of the Project Team or the chair of the research ethics committee?		

If the person answered no to any of these or are uncertain about any of these, do not seek written consent from the person. Instead, you (the project physiotherapist) will need to:

1) Ascertain if the person has either or both of the following:

- Advanced care directive (check that the research does not contravene this)
- A medical treatment decision maker

Informed consent will be sought from this medical treatment decision maker if there is an appointed medical treatment decision maker. If not, their informal carer will provide informed consent on behalf of the person. The informal carer is the first of the following with a close and continuing relationship with the person:

- The spouse or domestic partner
- The primary carer of the person
- The oldest adult child of the person

2) Explain the training program to the research decision maker (informal carer) and ensure their understanding

3) Seek informed consent from the research decision maker on behalf of the person before proceeding with the gait aid-training program.

In addition, for WA study participants (people with dementia/cognitive impairment) who are not able to provide consent, you (the project physiotherapist) will request the informal carer (*research decision maker*) to have the GAA form signed by their GP (*independent medical practitioner*). They will also take a letter to their GP (provided by the research team) which will explain to the GP about the research project and the reasons why they need to complete and sign the form.

The GAA form needs to be completed i.e. signed by the GP, you and the Lead Researcher (Fiona) before the gait-aid training can start.

Appendix B

Gait aid-training for people with dementia--Baseline (first home visit)

Start of Block: Demographics--Interview the person with dementia and the caregiver

This is the **baseline assessment** which occurs at the **first home visit.**

You will do the following at this home visit:

- Double check inclusion criteria if required
- Gain consent from the person with dementia/cognitive impairment (apply cognitive capacity checklist) and informal caregiver
- For persons with dementia/cognitive impairment that are unable to give their own consent:
    - For Vic, seek informal caregiver's consent on their behalf. Make sure he/she is a medical decision maker if there is an advanced care directive for this.
    - For WA, please follow the GAA consent procedure.
- Complete the relevant consent form for the person with dementia/cognitive impairment and informal caregiver
- Conduct an assessment for the participants before gait aid training (complete first half of the survey)
- Conduct gait aid training with the person with dementia/cognitive assessment
- Conduct an assessment after gait aid training (complete second half of the survey)
- Make your recommendation for home practice and instruct the informal caregiver if applicable. If not applicable, remove the gait aid and bring it back for the next home visit
- Give out falls and home practice diary to the participants and instruct them in how to complete them

This questionnaire includes assessments to be performed **BEFORE** and **AFTER** the gait aid training session:

Assessments **before** training:

- Demographics (interview the person with dementia or cognitive impairment and informal caregiver)
- Cognitive assessment (do a cognitive assessment with the person with dementia or cognitive impairment)
- Algorithm items (follow the items in the algorithm for assessment)
- Physical assessments (without a gait aid)

Assessment **after** training:

- Repeat physical assessments (with the recommended gait aid)
- Rate perception of safety and appropriateness of gait aid use by the physiotherapist, the person with dementia or cognitive impairment and the informal caregiver

Demographics--Interview the person with dementia or cognitive impairment and informal caregiver.

Please enter the ID of the person with dementia or cognitive impairment (using initials and date of birth). For example John Smith, date of birth 14/2/1950 will be JS14021950

____

Record the date of this home visit. For example 3/1/2021 will be 03012021

____

What is the age of the person with dementia or cognitive impairment? (please enter in years)

____

What is the gender of the person with dementia or cognitive impairment?

○ Male (1)

○ Female (2)

○ Other (3)

○ Prefer not to say (4)

Was the person formally diagnosed with dementia?

○ Yes (1)

○ No (2)

What type of dementia was the person diagnosed with?

- ○ Alzheimer's Disease (1)
- ○ Vascular dementia (2)
- ○ Lewy Body dementia (3)
- ○ Other dementia, please state (4) ____
- ○ Don't know/cannot remember (5)
- ○ The person has not been formally diagnosed with dementia (but meets the protocol definitions of having cognitive impairment) (6)

For how many years has the person been living with dementia (if the person was diagnosed with dementia)? Leave blank if has not been formally diagnosed

____

Who does the person with dementia or cognitive impairment live with? (Select all that apply)

- ▢ Spouse/partner (1)
- ▢ Children (2)
- ▢ Other relatives (3)
- ▢ Friend(s) (4)
- ▢ Lives alone (5)

What medical history does the person with dementia or cognitive impairment have? (Select all that apply)

- ☐ Anxiety (1)
- ☐ Arthritis (2)
- ☐ Cancer (3)
- ☐ Depression (4)
- ☐ Diabetes (5)
- ☐ Heart disease (6)
- ☐ Hip joint replacement (7)
- ☐ Kidney disease/renal failure (8)
- ☐ Knee joint replacement (9)
- ☐ Lung disease/respiratory disease (10)
- ☐ Parkinson's disease (11)
- ☐ Stroke (12)
- ☐ Other neurological disease (13)
- ☐ Visual impairment that are not correctable by glasses e.g. macular degeneration, glaucoma (14)
- ☐ Other, please state (15) ____

Physiotherapist to answer this question:
Can the person with dementia or cognitive impairment move around their home, including stopping and turning (observe)?

- ○ can do, appears steady (1)
- ○ can do but appears unsteady (2)
- ○ cannot do without assistance from someone else (3)

Can the person with dementia or cognitive impairment move outside their home (self i.e. person with dementia/cognitive impairment or carer report)?

- ○ can do, reported as steady and safe (1)
- ○ can do but reported as unsteady or unsafe (2)
- ○ cannot do without assistance from someone else (3)

Can the person with dementia or cognitive impairment walk up and down at least 3 stairs without a handrail or assistance from someone else (self i.e. person with dementia/cognitive impairment or carer report)?

- ○ can do without difficulty (1)
- ○ can do but with difficulty, or needs rail assistance (2)
- ○ cannot do without assistance from someone else (3)

Physiotherapist to answer this question:
Can the person with dementia or cognitive impairment bend and pick up an object from the floor without assistance from someone else (observe)?

○ can do without difficulty (1)

○ can do but with difficulty or unsteady (2)

○ cannot do without assistance from someone else (3)

Is the person with dementia or cognitive impairment currently engaging in a regular exercise or a physical activity program?

○ Yes, please describe what it is and how often, and overall exercise duration (minutes) for the past week (1) ____

○ No (2)

ALGORITHM ITEMS

Cognitive assessment:

Use cognitive test provided (Rowland Universal Dementia Assessment Scale-RUDAS) to assess the person with dementia or cognitive impairment.
N.B: Severity of cognitive impairment (RUDAS) 17-22=mild severity, 10-16= moderate severity, less than 10=severe dementia

The total score from the cognitive assessment is:

○ Mild severity, and the score is: (4)
____

○ Moderate severity, and the score is: (5)
____

○ Severe dementia, and the score is: (6)
____

Self (or carer's) report on ability of the person with dementia/cognitive impairment to remember new information

○ Can remember some information day to day (1)

○ Can remember some information over time, but limited day to day (2)

○ Cannot remember new information day to day (3)

Self (or carer's) rating of steadiness in walking, turning, going up and down stairs

○ Mostly steady, only occasional unsteadiness in turning or going up and down stairs (1)

○ Some unsteadiness on 1 to 2 of these activities (2)

○ Unsteady on all activities (3)

Did the person with dementia or cognitive impairment fall in the past 12 months?
A fall is defined as inadvertently coming to rest on the ground, floor or other lower level, excluding

intentional change in position to rest in furniture, wall or other objects.

◯ No falls (4)

◯ 1-3 falls (5)

◯ 4 or more falls (6)

Score the Elderly Falls Screening Tool (EFST). Refer to the training manual for scoring instructions.
The EFST score is:

____

Perform the TUG test (at a comfortable speed) without a gait aid. Allow a practice trial before the test.
Ask the person with dementia or cognitive impairment to stand up from a standard height (46cm) kitchen chair, walk three metres at their comfortable speed, turn, and return to the chair and sit down.

The time required (in seconds, to one decimal place) is:

____

Perform a walking test in a straight path (choose a corridor in the participant's home), then repeat the test asking the participant with dementia/cognitive impairment to count aloud backwards by 1's from 50 concurrently while walking.

◯ Able to walk and count aloud without slowing or stopping (1)

◯ Slows down when counting aloud but can keep walking (2)

◯ Unable to walk and counting aloud concurrently (i.e. needs to stop) (3)

Has the participant with dementia/cognitive impairment been taught by a physiotherapist in how to use a gait aid in the past and was it successful?

- ○ No (1)
- ○ Yes, was successful but the participant did not persist with gait aid use (2)
- ○ Yes but was unsuccessful (3)

Before training--Physical assessment: 4 metre walk test

Perform the 4 metre walk test (at a comfortable walking speed) **without a gait aid.** Allow a practice trial before the test.

- ☐ Time required (in seconds, to one decimal place) (1) ________________________________
- ☐ Gait speed (metre per second i.e. 4 divided by the time taken) (2) ________________________________
- ☐ No. of steps (3) ________________________________
- ☐ Step length (i.e. 4 m divided by the no. of steps) (4) ________________________________

Before training--Physical assessment: Figure of 8 walkway

Perform the Figure of 8 walkway test (at a comfortable speed) **without a gait aid.** Allow a practice trial before the test.

Instructions to the person with dementia or cognitive impairment:

- Stand between the 2 cones (placed 5 feet apart i.e. 1.5 m), facing in one direction (the starting/stopping point is not marked "… in order to avoid influencing the movement planning for the task.")
- Begin walking at your <u>usual pace</u> when ready (the person chooses which direction to go around the cones)
- Stop when you return to the start position.

Instructions to the therapist:

- Timing: begin timing with the first step, and stop timing when the person with dementia assumes a side-by-side stance of the feet back at the start position.
- Steps: count the number of steps taken.
- Accuracy: "The 2-ft (0.6 m) surround test boundary was not marked on the course. The tester determined the 2-ft (0.6 m) boundary area for the test setup and the relationships of the boundary to the testing space (e.g. distance from the hallway walls, floor markings, or landmarks) prior to testing, and estimated whether the test was completed within the boundary by comparison with the tester's mental map of the testing space."

☐ Time required (in seconds, to one decimal place) (1)

____

☐ No. of steps (2) ____

☐ Accuracy: does the person stay within the unmarked 2-foot (0.6 m) surround boundary of the cones? Write Yes or No response only. (3)

____

Before training-repeat the Figure of 8 walkway test (at a comfortable speed) **without a gait aid AND with a dual cognitive task.** Allow a practice trial before the test.

Instructions to the person with dementia or cognitive impairment:

- Stand between the 2 cones (placed 5 feet apart i.e. 1.5 m), facing in one direction (the starting/stopping point is not marked "… in order to avoid influencing the movement planning for the task.")
- Begin walking at your usual pace when ready (the person chooses which direction to go around the cones) and count backwards by 1's from 50 whilst walking.
- Stop when you return to the start position.

Instructions to the therapist:

- Timing: begin timing with the first step, and stop timing when the person with dementia assumes a side-by-side stance of the feet back at the start position.
- Steps: count the number of steps taken.
- Accuracy: "The 2-ft (0.6 m) surround test boundary was not marked on the course. The tester determined the 2-ft (0.6 m) boundary area for the test setup and the relationships of the boundary to the testing space (e.g. distance from the hallway walls, floor markings, or landmarks) prior to testing, and estimated whether the test was completed within the boundary by comparison with the tester's mental map of the testing space."

☐ Time required (in seconds, to one decimal place) (1)

____

☐ No. of steps (2) ____

☐ Accuracy: does the person stay within the unmarked 2-foot (0.6 m) surround boundary of the cones? Write Yes or No response only. (3)

____

☐ Unable to perform this test (i.e. the person cannot walk and count backwards concurrently- needs to stop) (4)

Having completed this assessment (before starting gait aid training) – what is your (the physiotherapist) perception of the likelihood of this person with dementia/cognitive impairment

being able to achieve safe gait aid training at the end of this 6 week training? (0=extremely unlikely, 5=moderate likely and 10=extremely likely)

	0 (1)	1 (2)	2 (3)	3 (4)	4 (5)	5 (6)	6 (7)	7 (8)	8 (9)	9 (10)	10 (11)
Perception of likelihood of the person being able to achieve safe gait aid training at the end of the 6 week training program (1)	○	○	○	○	○	○	○	○	○	○	○

Do you consider it safe and acceptable to the person with dementia and their caregiver to proceed with the 6 week gait aid training program?

○ Yes. Proceed to the next question (1)

○ No. Finish here, thank the person with dementia/cognitive impairment and their carer for their time. Clarify for them the reasons you will not be proceeding with the gait aid training program. State the reason why. (2) ____

Perform gait aid training with the participant with dementia/cognitive impairment.

The following questions are to be answered after the gait aid training session.

What gait aid have you used to provide training for the person with dementia or cognitive impairment?

- ○ Walking stick/cane (1)
- ○ 4-point stick (2)
- ○ Pick-up frame (3)
- ○ Wheelie walking frame (4)
- ○ Other, please state (5) ____

State the main reason(s) for selecting this gait aid.

____

After training--Perception rating of safety and appropriateness of gait aid use.

Ask the **participant** with dementia or cognitive impairment after the gait aid training session to rate the following statements:

	Strongly agree (1)	Somewhat agree (2)	Neither agree nor disagree (3)	Somewhat disagree (4)	Strongly disagree (5)
Walking with (state the type of gait aid) has improved my walking (1)	○	○	○	○	○
Walking with (state the type of gait aid) has made me feel safe (2)	○	○	○	○	○
Walking with (state the type of gait aid) feels right for me (3)	○	○	○	○	○

Ask the **participant with dementia or cognitive impairment** to provide comments for the above responses. Record the comments here.

____

Ask the **informal caregiver** after the gait aid training session to rate the following statements:

	Strongly agree (1)	Somewhat agree (2)	Neither agree nor disagree (3)	Somewhat disagree (4)	Strongly disagree (5)
The gait aid has improved the participant's steadiness in walking (1)	○	○	○	○	○
The gait aid has improved the participant's safety in walking (2)	○	○	○	○	○
Using the gait aid is appropriate for the participant (3)	○	○	○	○	○

Ask the **informal caregiver** to provide comments for the above responses. Record the comments here.

____

Give **your opinion (i.e. physiotherapist)** after the gait aid training session to rate the following statements:

	Strongly agree (1)	Somewhat agree (2)	Neither agree nor disagree (3)	Somewhat disagree (4)	Strongly disagree (5)
The gait aid has improved the participant's steadiness in walking (1)	○	○	○	○	○
The gait aid has improved the participant's safety in walking (2)	○	○	○	○	○
Using the gait aid is appropriate for the participant (3)	○	○	○	○	○

Give your (i.e. physiotherapist) comments for the above responses. Record the comments here.

____

After training--Physical assessment: 4 metre walk test

Perform the 4 metre walk test (at a comfortable walking speed) **with the recommended gait aid.** Allow a practice trial before the test.

- ☐ Time required (in seconds, to one decimal place) (1) ____

- ☐ Gait speed (metre per second i.e. 4 divided by the time taken) (2) ____

- ☐ No. of steps (3) ____

- ☐ Step length (i.e. 4 m divided by the no. of steps) (4) ____

After training--Physical assessment: Timed Up and Go (TUG) Test

Perform the TUG test (at a comfortable speed) **with the recommended gait aid**. Allow a practice trial before the test.

Ask the person with dementia or cognitive impairment to stand up from a standard height kitchen chair (46cm), walk three metres at their comfortable speed, turn, and return to the chair and sit down.

- ☐ Time required (in seconds, to one decimal place) (1) ____

After training--Physical assessment: Figure of 8 walkway

Perform the Figure of 8 walkway test (at a comfortable speed) **with the recommended gait aid.** Allow a practice trial before the test.

Instructions to person with dementia or cognitive impairment:

- Stand between the 2 cones (placed 5 feet apart i.e. 1.5 m), facing in one direction (the starting/stopping point is not marked "… in order to avoid influencing the movement planning for the task.")
- Begin walking at your usual pace when ready (the person chooses which direction to go around the cones)
- Stop when you return to the start position.

Instructions to the therapist:

- Timing: begin timing with the first step, and stop timing when the person with dementia assumes a side-by-side stance of the feet back at the start position.
- Steps: count the number of steps taken.
- Accuracy: "The 2-ft (0.6 m) surround test boundary was not marked on the course. The tester determined the 2-ft (0.6 m) boundary area for the test setup and the relationships of the boundary to the testing space (e.g. distance from the hallway walls, floor markings, or landmarks) prior to testing, and estimated whether the test was completed within the boundary by comparison with the tester's mental map of the testing space."

☐ Time required (in seconds, to one decimal place) (1)
____

☐ No. of steps (2) ____

☐ Accuracy: does the person stay within the unmarked 2-foot (0.6 m) surround boundary of the cones? Write Yes or No response only. (3)
____

After training-repeat the Figure of 8 walkway test (at a comfortable speed) **with the recommended gait aid AND with a dual cognitive task.** Allow a practice trial before the test.

Instructions to person with dementia or cognitive impairment:

- Stand between the 2 cones (placed 5 feet apart i.e. 1.5 m), facing in one direction (the starting/stopping point is not marked "… in order to avoid influencing the movement planning for the task.")
- Begin walking at your usual pace when ready (the person chooses which direction to go around the cones) and count backwards by 1's from 50 whilst walking.
- Stop when you return to the start position.

Instructions to the therapist:

- Timing: begin timing with the first step, and stop timing when the person with dementia assumes a side-by-side stance of the feet back at the start position.
- Steps: count the number of steps taken.
- Accuracy: "The 2-ft (0.6 m) surround test boundary was not marked on the course. The tester determined the 2-ft (0.6 m) boundary area for the test setup and the relationships of the boundary to the testing space (e.g. distance from the hallway walls, floor markings, or landmarks) prior to testing, and estimated whether the test was completed within the boundary by comparison with the tester's mental map of the testing space."

☐ Time required (in seconds, to one decimal place) (1)

____

☐ No. of steps (2) ____

☐ Accuracy: does the person stay within the unmarked 2-foot (0.6 m) surround boundary of the cones? Write Yes or No response only. (3)

____

☐ Unable to perform this test (i.e. the person cannot walk and count backwards concurrently- needs to stop) (4)

Ask the person with dementia or cognitive impairment:

How did the person with dementia or cognitive impairment find the training session with the physiotherapist?

Rate on a 0-10 point scale (0=extremely difficult, cannot manage, 10=no difficulties at all, can manage it very well) and provide comments.

____

Ask the informal caregiver:
How did the informal caregiver find the training session the person with dementia or cognitive impairment had with the physiotherapist?
Rate on a 0-10 point scale (0=extremely difficult, cannot manage, 10=no difficulties at all, can manage it very well) and provide comments.

____

What recommendations have you provided to the person with dementia or cognitive impairment and their informal caregiver at the end of the session? (based on the responses of the person with dementia/cognitive impairment, objective gait/mobility measurements and your clinical judgement)

- ○ Leave the gait aid with the person with dementia and informal caregiver to practise at home, please record the instruction here i.e. nature, content, frequency and duration of home practice (1) ____

- ○ Remove the gait aid at the end of the session (informal caregiver does not feel confident to supervise gait aid practice with the person with dementia). Participant only practices with the physiotherapist. (2)

- ○ Remove the gait aid at the end of the session (the physiotherapist does not feel it is safe to leave the gait aid for home practice). Participant only practices with the physiotherapist. (3)

Please explain the strategies that you have used to help the person with dementia/cognitive impairment to learn the safe and correct use of the gait aid during this session (e.g. memory aids, cues, structuring of practice tasks, environment etc) Select all that apply.

- [ ] Constant pattern of practice (1)
- [ ] Constant pattern of instruction (2)
- [ ] Memory aid (3)
- [ ] Verbal cues (4)
- [ ] Visual cues (5)
- [ ] Start with non-complex environment, then progress to more complex environment (6)
- [ ] Avoid dual tasking during initial learning e.g. minimise talking from the person, talking to the person or stop them from walking with the gait aid when they or you want to talk (7)
- [ ] Other, please provide details (can include the strategies provided by the physiotherapist to the informal caregiver) (8) ____

Do you think the person with dementia/cognitive impairment has successfully demonstrated the safe and correct use of gait aid in this session?

- ○ Yes (1)
- ○ Partially (2)
- ○ No (3)

Do you (physiotherapist) have any further comments regarding the gait aid training program?

____

Please provide your (physiotherapist) name so that the research team can contact you in case of queries.

____

Thank you for completing the data collection survey. Please move to the next page to submit the survey online.

End of Block: Demographics--Interview the person with dementia and the caregiver

Appendix C

Rowland Universal Dementia Assessment Scale (RUDAS)

Refer to the RUDAS Administration and Scoring guide

https://metronorth.health.qld.gov.au/tpch/wp-content/uploads/sites/3/2017/09/rudas.pdf

R U D A S

The Rowland Universal Dementia Assessment Scale: A Multicultural Cognitive Assessment Scale.
(Storey, Rowland, Basic, Conforti & Dickson, 2004). International Psychogeriatrics, 16 (1), 13-31

Date: ____/____/____ Patient **Name:** ____

Item		Max Score

Memory
1. (Instructions) I want you to imagine that we are going shopping. Here is a list of grocery items. I would like you to remember the following items which we need to get from the shop. When we get to the shop in about 5 mins. time I will ask you what it is that we have to buy. You must remember the list for me.
Tea, Cooking Oil, Eggs, Soap Please repeat this list for me (ask person to repeat the list 3 times). (If person did not repeat all four words, repeat the list until the person has learned them and can repeat them, or, up to a maximum of five times.)

Visuospatial Orientation
2. I am going to ask you to identify/show me different parts of the body. *(Correct = 1)*. Once the person correctly answers 5 parts of this question, do not continue as the maximum score is 5.

(1) show me your right foot 1
(2) show me your left hand 1
(3) with your right hand touch your left shoulder 1
(4) with your left hand touch your right ear 1
(5) which is (indicate/point to) my left knee 1
(6) which is (indicate/point to) my right elbow 1
(7) with your right hand indicate/point to my left eye 1
(8) with your left hand indicate/point to my left foot 1

..../5

Praxis
3. I am going to show you an action/exercise with my hands. I want you to watch me and copy what I do. Copy me when I do this . . . (One hand in fist, the other palm down on table - alternate simultaneously.) Now do it with me: Now I would like you to keep doing this action at this pace until I tell you to stop - approximately 10 seconds. (Demonstrate at moderate walking pace).
Score as:
Normal = 2 *(very few if any errors; self-corrected, progressively better; good maintenance; only very slight lack of synchrony between hands)*
Partially Adequate = 1 *(noticeable errors with some attempt to self-correct; some attempt at maintenance; poor synchrony)*
Failed = 0 *(cannot do the task; no maintenance; no attempt whatsoever)*

..../2

Visuoconstructional Drawing
4. Please draw this picture exactly as it looks to you (Show cube on back of page). *(Yes = 1)*
Score as:
(1) Has person drawn a picture based on a square? 1
(2) Do all internal lines appear in person's drawing? 1

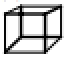

(3) Do all external lines appear in person's drawing? 1

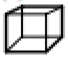

..../3

Judgment
5. You are standing on the side of a busy street. There is no pedestrian crossing and no traffic lights. Tell me what you would do to get across to the other side of the road **safely**. (If person gives incomplete response that does not address both parts of answer, use prompt: "Is there anything else you would do?") Record exactly what patient says and circle all parts of response which were prompted.
....
....

Score as:
Did person indicate that they would look for traffic? *(YES = 2; YES PROMPTED = 1; NO = 0)* 2
Did person make any additional safety proposals? *(YES = 2; YES PROMPTED = 1; NO = 0)* 2

..../4

**Memory Recall**<br>1. (Recall) We have just arrived at the shop. Can you remember the list of groceries we need to buy? (Prompt: If person cannot recall any of the list, say "The first one was 'tea'." *(Score 2 points each for any item recalled which was not prompted – use only 'tea' as a prompt.)*<br>*Tea*<br>*Cooking Oil*<br>*Eggs*<br>*Soap*	…….2<br>…….2<br>…….2<br>…….2	**…./8**
**Language**<br>6. I am going to time you for one minute. In that one minute, I would like you to tell me the names of as many different animals as you can. We'll see how many different animals you can name in one minute. (Repeat instructions if necessary). Maximum score for this item is 8. If person names 8 new animals in less than one minute there is no need to continue.<br>1. ……………………………… 5. ………………………………<br>2. ……………………………… 6. ………………………………<br>3. ……………………………… 7. ………………………………<br>4. ……………………………… 8. ………………………………		**…./8**
**TOTAL SCORE =**		**/30**

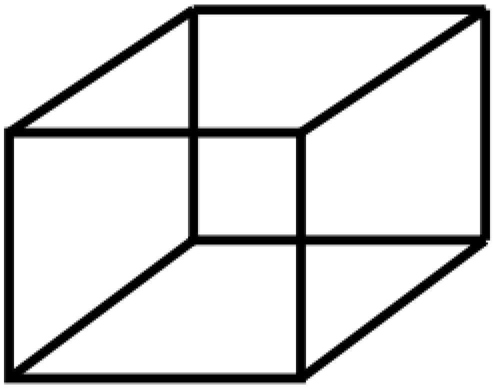

Appendix D

Decision aid tool for Physiotherapists, Nurses and Occupational Therapists: Gait aid use for people with dementia

**Item**	**1 (low risk)**	**2 (medium risk)**	**3 (high risk)**	**Score (rate each item i.e. give 1 point for low risk, 2 points for medium risk and 3 points for high risk)**
Severity of cognitive impairment (Rowland Universal Dementia Assessment Scale - RUDAS)*ϕ	RUDAS score (mild severity) 17-22	RUDAS score (moderate severity) 10-16	RUDAS score (severe dementia) <10	
Self (or carer's) report on ability to remember new information	Can remember some information day to day	Can remember some information over time, but limited day to day	Cannot remember new information day to day	
Self (or carer's) rating of steadiness in walking, turning, going up and down stairs	Mostly steady, only occasional unsteadiness on turning / stairs	Some unsteadiness on 1-2 of these activities	Unsteady on all activities	
Self-reported (or carer's reported) number of falls in the preceding 12 months	Nil	1-3	4 or more	
Elderly Falls Screening Tool (EFST) (refer to Table 1 below)	EFST 0-1	EFST 2-3	EFST 4-5	
Mobility – Timed Up and Go test[¥]	≤10 seconds	>10—15 seconds	>15 seconds	
Able to dual task (talk while walking)	Able to walk and count aloud backwards by 1's from 50 without slowing or stopping	Slows down when counting aloud backwards by 1's from 50 while walking but can keep counting aloud	Unable to walk and count aloud backwards by 1's from 50 concurrently – needs to stop	
Previous trial/s with a gait aid with **therapist involvement**	Nil	Yes, but did not persist with gait aid use	Yes, but was not successful	
**TOTAL SCORE (add scores for all items)**				

* Alternative assessments of cognition can be used – but will need to use appropriate cut-off scores for mild, moderate and severe cognitive impairment

e.g. Mini Mental State Examination (MMSE) range 0-30, cut-off scores for mild cognitive impairment is 21-24, moderate cognitive impairment is 10-20, severe cognitive impairment is 0-9 (https://www.caring.com/examinations/what-does-your-mmse-score-mean)

Montreal Cognitive Assessment (MoCA) range 0-30, cut-off scores for mild cognitive impairment is 19-25. Cut-offs for dementia detection is dependent on race and education attainment (Milani et al. 2018 doi: 10.1016/j.dadm.2018.09.003)

ϕ RUDAS classification from Summary recommendations from Dementia Driving Guidelines (2014)

¥ https://www.sralab.org/rehabilitation-measures/timed-and-go

Indicator of likely success of gait aid training:

Score	Likelihood of being able to safely learn gait aid use with training / practice
≤10	Good
11-17	Intermediate
≥ 18	Poor

NOTE – this scoring is an aid for clinical decision making. The health professional may use additional information and their clinical judgement combined with the data from this decision support tool, to make a final decision about providing a client with dementia a training trial for using a gait aid.

Table 1. Procedures for Elderly Fall Screening Test (EFST)

Part 1: Self-reported fall history

After defining a fall, ask “how many times in the past year did you fall?” (you can use the answer from the item above)

- falls, score=0
- 2+ falls, score=1

For those who report any fall in the past year, ask “Did you injure yourself?”

- No injury from any fall, score=0
- Any injury (soft tissue, fracture), score=1
- For all respondents, ask “How often does it happen to you that you think you are about to fall, but manage to grab something and then don’t fall?”
- Never or rarely report “near falls”, score=0
- Frequently or occasionally report “near falls”, score=1

Part 2: Observation on gait patterns

Respondent is asked to walk at normal pacing speed over a 4m distance

Walking speed is recorded with a stop watch

- If walking speed is faster than or equal to 0.5m/s, score=0
- If walking speed is slower than 0.5m/s, score=1
- Gait style is observed and recorded
- If gait is even, straight and feet are raised with each step, score=0
- If gait is uneven, shuffling, on a wide base or unsteady, score=1

Add the scores from each question in part 1 and 2.

Appendix E

4 metre walk test

Equipment Required

- Stopwatch
- A clear pathway with set distance (4 metres in length with an extra 1 metre at either end)
- Tape measure

Set-Up

- Measure and mark a 4-metre walkway
- Add a mark 1 metre before and 1 metre after the 4 metre walkway

Instructions

- The individual walks without assistance for 4 metres
- Assistive devices may be used, but must be kept consistent and documented for each test
- Start timing when the toes pass the start of 4-metre mark
- Stop timing when the toes pass the end of 4-metre mark
- Test at a comfortable walking speed
- Perform one practice trial before the test
- Record time required in seconds
- Count number of steps

Patient Instructions

- "I will say ready, set, go. When I say go, walk at your normal comfortable speed until I say stop"

Appendix F

Time Up and Go test

Equipment Required

- Standard height chair (46 cm)
- Stop watch
- Tape measure

Set-Up

- Measure and mark a 3-metre walkway
- Place the chair at the start of the 3-metre walkway

Instructions

- The individual sits in the chair with his/her back against the chair back
- Assistive devices may be used, but must be kept consistent and documented for each test
- Start timing at the instruction “go”
- Stop timing when the person is seated
- Test at a comfortable walking speed
- Perform one practice trial before the scoring
- Record time required in seconds

Patient Instructions

- “I will say ready, set, go. When I say go, rise up from the chair, walk at your normal comfortable speed to the 3-metre mark, turn and walk back to the chair and sit down”

Appendix G

Figure of 8 walkway

Equipment Required

- 2 cones
- Stop watch
- Tape measure

Set-Up

- Place 2 cones 1.5m apart

Instructions

- Assistive devices may be used, but must be kept consistent and documented for each test
- Start timing with the first step
- Stop timing when the person assumes a side-by-side stance of the feet back at the start position
- Test at a comfortable walking speed
- Count the number of steps taken
- Record the time required in seconds
- Accuracy: The 0.6 m surround test boundary was not marked on the course. The tester determined the 0.6 m boundary area for the test setup and the relationships of the boundary to the testing space (e.g. distance from the hallway walls, floor markings, or landmarks) prior to testing, and estimated whether the test was completed within the boundary by comparison with the tester's mental map of the testing space
- Perform one practice trial before the scoring

Patient Instructions

- "Stand between the 2 cones, facing in one direction. Begin walking at your usual pace when ready (the person chooses which direction to go around the cones). Stop when you return to the start position.

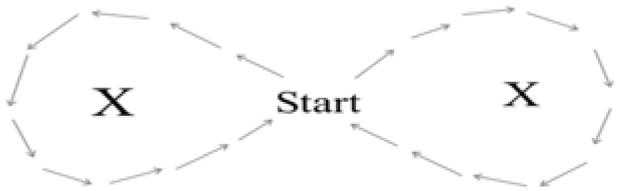

The Figure-8 Walk Test
(Adapted from Hess et al., 2010)

Figure of 8 walkway and dual cognitive task

Equipment required, set-up, instructions as per above

Patient Instructions

"Stand between the 2 cones, facing in one direction. Begin walking at your usual pace when ready (the person chooses which direction to go around the cones) and **count backwards by 1's from 50 whilst walking**. Stop when you return to the start position.

Appendix H

Gait aid-training for people with dementia--2nd week (second home visit)

Start of Block: Demographics--Interview the person with dementia and the caregiver

This is the **second assessment** which occurs at the **second home visit** at the second week.

You will do the following at this home visit:

- Conduct an assessment for the participants before gait aid training (complete first half of the survey)
- Conduct gait aid training with the person with dementia/cognitive impairment
- Conduct an assessment for the participants after gait aid training (complete second half of survey)
- Make your recommendation for home practice and instruct the informal caregiver if applicable. If not applicable, remove the gait aid and bring it back for the next home visit.

This questionnaire includes assessments to be performed **BEFORE and AFTER** the gait aid training session:

Assessments **before** training:

- Number of falls and its associated factors in the last week
- Adverse events in the last week associated with home practice if applicable
- Adherence to home practice sessions carried out by the person with dementia or cognitive impairment and informal caregiver in the last week

Assessment **after** training:

- Rate perception of safety and appropriateness of gait aid use by the physiotherapist, the person with dementia or cognitive impairment and the informal caregiver

---

Please enter the ID of the person with dementia or cognitive impairment (using initials and date of birth). For example John Smith, date of birth 14/2/1950 will be JS14021950

____

Record the date of this home visit. For example 3/1/2021 will be 03012021

____

Did the person with dementia or cognitive impairment fall in the last week?
A fall is defined as "inadvertently coming to rest on the ground, floor or other lower level, excluding intentional change in position to rest in furniture, wall or other objects.

Please enter the number of falls i.e. 0 for no falls, 1 for 1 fall etc. Leave blank if unknown/cannot remember.

____

If a fall happened in the last week, please give details in the item below: (put all information into each box provided respectively if there were multiple falls and/or injuries from falls i.e. format: fall 1 circumstance=xxx, fall 2=xxx etc, injury 1=xxx, injury 2=xxx)

- ☐ The circumstance of the fall(s) was: (1) ____

- ☐ The injury from the fall(s) was: enter none if no injury (2) ____

Has the gait aid been left for home practice between sessions?

- ○ Yes (1)
- ○ No (2)

If a gait aid was left for home practice, was the person using the gait aid when they fell?

○ Yes. Ask the caregiver if they thought the gait aid might have contributed to the fall--if yes, please give details (1) ____

○ No (2)

○ No fall (3)

Did the person with dementia or cognitive impairment and informal caregiver adhere to home practice in the last week as per the physiotherapist's recommendations?

○ Yes. Full adherence to the frequency and duration of practice (1)

○ No. Partial adherence to the frequency and duration of practice, please state the reasons why. (2) ____

○ No. No practice at all, please state the reasons why. (3) ____

○ No home practice was recommended for the last week (4)

Ask the person with dementia or cognitive impairment:
How did the person with dementia or cognitive impairment find the home practice session (if home practice was applicable)?
Rate on a 0-10 point scale (0=extremely difficult, cannot manage, 10=no difficulties at all, can manage it very well) and provide comments.

____

Ask the informal caregiver:
How did the informal caregiver find the home practice session (if home practice was applicable)?
Rate on a 0-10 point scale (0=extremely difficult, cannot manage, 10=no difficulties at all, can manage it very well) and provide comments.

____

Perform gait aid training with the participant with dementia/cognitive impairment.
The following questions are to be answered after gait aid training.

What gait aid have you used to provide training for the person with dementia or cognitive impairment?

- ○ Walking stick/cane (1)
- ○ 4-point stick (2)
- ○ Pick-up frame (3)
- ○ Wheelie walking frame (4)
- ○ Other, please state (5) ____

Has this changed from the previous session?

- ○ Yes. Please explain why and the factors you considered in changing the gait aid. (1) ____
- ○ No (2)

After training--Perception rating of safety and appropriateness of gait aid use.

Ask the **participant** with dementia or cognitive impairment after the gait aid training session to rate the following statements:

	Strongly agree (1)	Somewhat agree (2)	Neither agree nor disagree (3)	Somewhat disagree (4)	Strongly disagree (5)
Walking with (state the type of gait aid) has improved my walking (1)	○	○	○	○	○
Walking with (state the type of gait aid) has made me feel safe (2)	○	○	○	○	○
Walking with (state the type of gait aid) feels right for me (3)	○	○	○	○	○

Ask the **participant with dementia or cognitive impairment** to provide comments for the above responses. Record the comments here.

____

Ask the **informal caregiver** after the gait aid training session to rate the following statements:

	Strongly agree (1)	Somewhat agree (2)	Neither agree nor disagree (3)	Somewhat disagree (4)	Strongly disagree (5)
The gait aid has improved the participant's steadiness in walking (1)	○	○	○	○	○
The gait aid has improved the participant's safety in walking (2)	○	○	○	○	○
Using the gait aid is appropriate for the participant (3)	○	○	○	○	○

Ask the **informal caregiver** to provide comments for the above responses. Record the comments here.

____

Give **your opinion** (i.e. physiotherapist) after the gait aid training session to rate the following statements:

	Strongly agree (1)	Somewhat agree (2)	Neither agree nor disagree (3)	Somewhat disagree (4)	Strongly disagree (5)
The gait aid has improved the participant's steadiness in walking (1)	○	○	○	○	○
The gait aid has improved the participant's safety in walking (2)	○	○	○	○	○
Using the gait aid is appropriate for the participant (3)	○	○	○	○	○

Give your (i.e. physiotherapist) comments for the above responses. Record the comments here.

____

Ask the person with dementia or cognitive impairment:
How did the person with dementia or cognitive impairment find the training session with the physiotherapist?
Rate on a 0-10 point scale (0=extremely difficult, cannot manage, 10=no difficulties at all, can manage it very well) and provide comments.

____

Ask the informal caregiver:
How did the informal caregiver find the training session the person with dementia or cognitive

impairment had with the physiotherapist? Rate on a 0-10 point scale (0=extremely difficult, cannot manage, 10=no difficulties at all, can manage it very well) and provide comments.

____

What recommendations have you provided to the person with dementia or cognitive impairment and their informal caregiver at the end of the session? (based on the responses of the person with dementia/cognitive impairment, objective gait/mobility measurements and your clinical judgement)

○ Leave the gait aid with the person with dementia and informal caregiver to practise at home, please record the instruction here i.e. nature, content, frequency and duration of home practice (1) ____

○ Remove the gait aid at the end of the session (informal caregiver does not feel confident to supervise gait aid practice with the person with dementia). Participant only practices with the physiotherapist. (2)

○ Remove the gait aid at the end of the session (the physiotherapist does not feel it is safe to leave the gait aid for home practice). Participant only practices with the physiotherapist (3)

Please explain the strategies that you have used to help the person with dementia/cognitive impairment to learn the safe and correct use of the gait aid during this session (e.g. memory aids, cues, structuring of practice tasks, environment etc) Select all that apply.

- [ ] Constant pattern of practice (1)
- [ ] Constant pattern of instruction (2)
- [ ] Memory aid (3)
- [ ] Verbal cues (4)
- [ ] Visual cues (5)
- [ ] Start with non-complex environment, then progress to more complex environment (6)
- [ ] Avoid dual tasking during initial learning e.g. minimise talking from the person, talking to the person or stop them from walking with the gait aid when they or you want to talk (7)
- [ ] Other, please provide details (can include the strategies provided by the physiotherapist to the informal caregiver) (8) ____

Do you think the person with dementia/cognitive impairment has successfully demonstrate the safe and correct use of gait aid in this session?

- ○ Yes (1)
- ○ Partially (2)
- ○ No (3)

Do you (physiotherapist) have any further comments regarding the gait aid training program?

____

Please provide your (physiotherapist) name so that the research team can contact you in case of queries.

____

Thank you for completing the data collection survey. Please move to the next page to submit the survey online.

End of Block: Demographics--Interview the person with dementia and the caregiver

Appendix I

Gait aid-training for people with dementia--3rd week (third home visit)

Start of Block: Demographics--Interview the person with dementia and the caregiver

This is the **third assessment** which occurs at the **third home visit** at the third week.

You will do the following at this home visit:

- Conduct an assessment for the participants before gait aid training (complete first half of the survey)
- Conduct gait aid training with the person with dementia/cognitive impairment
- Conduct an assessment for the participants after gait aid training (complete second half of survey)
- Make your recommendation for home practice and instruct the informal caregiver if applicable. If not applicable, remove the gait aid and bring it back for the next home visit.

This questionnaire includes assessments to be performed **BEFORE and AFTER** the gait aid training session

Assessments **before** training:

- Number of falls and its associated factors in the last week
- Adverse events in the last week associated with home practice if applicable
- Adherence to home practice sessions carried out by the person with dementia or cognitive impairment and informal caregiver in the last week

Assessment **after** training:

- Rate perception of safety and appropriateness of gait aid use by the physiotherapist, the person with dementia or cognitive impairment and the informal caregiver

---

Please enter the ID of the person with dementia or cognitive impairment (using initials and date of birth). For example John Smith, date of birth 14/2/1950 will be JS14021950

____

Record the date of this home visit. For example 3/1/2021 will be 03012021

____

Did the person with dementia or cognitive impairment fall in the last week?
A fall is defined as "inadvertently coming to rest on the ground, floor or other lower level, excluding intentional change in position to rest in furniture, wall or other objects.

Please enter the number of falls i.e. 0 for no falls, 1 for 1 fall etc. Leave blank if unknown/cannot remember.

____

If a fall happened in the last week, please give details in the item below: (put all information into each box provided respectively if there were multiple falls and/or injuries from falls i.e. format: fall 1 circumstance=xxx, fall 2=xxx etc, injury 1=xxx, injury 2=xxx)

- ▢ The circumstance of the fall(s) was: (1) ____

- ▢ The injury from the fall(s) was: enter none if no injury (2) ____

Has the gait aid been left for home practice between sessions?

- ○ Yes (1)
- ○ No (2)

If a gait aid was left for home practice, was the person using the gait aid when they fell?

- ◯ Yes. Ask the caregiver if they consider that the gait aid might have contributing to the fall--if yes, please give details (1) ____
- ◯ No (2)
- ◯ No fall (3)

Did the person with dementia or cognitive impairment and informal caregiver adhere to home practice in the last week as per the physiotherapist's recommendations?

- ◯ Yes. Full adherence to the frequency and duration of practice (1)
- ◯ No. Partial adherence to the frequency and duration of practice, please state the reasons why. (2) ____
- ◯ No. No practice at all, please state the reasons why. (3) ____
- ◯ No home practice was recommended for the last week (4)

Ask the person with dementia or cognitive impairment: How did the person with dementia or cognitive impairment find the home practice session (if home practice was applicable)? Rate on a 0-10 point scale (0=extremely difficult, cannot manage, 10=no difficulties at all, can manage it very well) and provide comments.

____

Ask the informal caregiver: How did the informal caregiver find the home practice session (if home practice was applicable)? Rate on a 0-10 point scale (0=extremely difficult, cannot manage, 10=no difficulties at all, can manage it very well) and provide comments.

____

Perform gait aid training with the participant with dementia/cognitive impairment.
The following questions are to be answered after gait aid training.

What gait aid have you used to provide training for the person with dementia or cognitive impairment?

- ◯ Walking stick/cane (1)
- ◯ 4-point stick (2)
- ◯ Pick-up frame (3)
- ◯ Wheelie walking frame (4)
- ◯ Other, please state (5) ____

Has this changed from the previous session?

- ◯ Yes. Please explain why and the factors you considered in changing the gait aid. (1) ____
- ◯ No (2)

After training--Perception rating of safety and appropriateness of gait aid use.

Ask the **participant** with dementia or cognitive impairment after the gait aid training session to rate the following statements:

	Strongly agree (1)	Somewhat agree (2)	Neither agree nor disagree (3)	Somewhat disagree (4)	Strongly disagree (5)
Walking with (state the type of gait aid) has improved my walking (1)	○	○	○	○	○
Walking with (state the type of gait aid) has made me feel safe (2)	○	○	○	○	○
Walking with (state the type of gait aid) feels right for me (3)	○	○	○	○	○

Ask the **participant with dementia or cognitive impairment** to provide comments for the above responses. Record the comments here.

____

Ask the **informal caregiver** after the gait aid training session to rate the following statements:

	Strongly agree (1)	Somewhat agree (2)	Neither agree nor disagree (3)	Somewhat disagree (4)	Strongly disagree (5)
The gait aid has improved the participant's steadiness in walking (1)	○	○	○	○	○
The gait aid has improved the participant's safety in walking (2)	○	○	○	○	○
Using the gait aid is appropriate for the participant (3)	○	○	○	○	○

Ask the **informal caregiver** to provide comments for the above responses. Record the comments here.

____

Give **your opinion (i.e. physiotherapist)** after the gait aid training session to rate the following statements:

	Strongly agree (1)	Somewhat agree (2)	Neither agree nor disagree (3)	Somewhat disagree (4)	Strongly disagree (5)
The gait aid has improved the participant's steadiness in walking (1)	○	○	○	○	○
The gait aid has improved the participant's safety in walking (2)	○	○	○	○	○
Using the gait aid is appropriate for the participant (3)	○	○	○	○	○

Give your (i.e. physiotherapist) comments for the above responses. Record the comments here.

____

Ask the person with dementia or cognitive impairment:
How did the person with dementia or cognitive impairment find the training session with the physiotherapist?
Rate on a 0-10 point scale (0=extremely difficult, cannot manage, 10=no difficulties at all, can manage it very well) and provide comments.

____

Ask the informal caregiver:
How did the informal caregiver find the training session the person with dementia or cognitive impairment had with the physiotherapist?

Rate on a 0-10 point scale (0=extremely difficult, cannot manage, 10=no difficulties at all, can manage it very well) and provide comments.

____

What recommendations have you provided to the person with dementia or cognitive impairment and their informal caregiver at the end of the session? (based on the responses of the person with dementia/cognitive impairment and objective gait/mobility measurements)

○ Leave the gait aid with the person with dementia and informal caregiver to practise at home, please record the instruction here i.e. nature, content, frequency and duration of home practice (1) ____

○ Remove the gait aid at the end of the session (informal caregiver does not feel confident to supervise gait aid practice with the person with dementia). Participant only practices with the physiotherapist. (2)

○ Remove the gait aid at the end of the session (the physiotherapist does not feel it is safe to leave the gait aid for home practice). Participant only practices with the physiotherapist. (3)

Please explain the strategies that you have used to help the person with dementia/cognitive impairment to learn the safe and correct use of the gait aid during this session (e.g. memory aids, cues, structuring of practice tasks, environment etc) Select all that apply.

- ▢ Constant pattern of practice (1)
- ▢ Constant pattern of instruction (2)
- ▢ Memory aid (3)
- ▢ Verbal cues (4)
- ▢ Visual cues (5)
- ▢ Start with non-complex environment, then progress to more complex environment (6)
- ▢ Avoid dual tasking during initial learning e.g. minimise talking from the person, talking to the person or stop them from walking with the gait aid when they or you want to talk (7)
- ▢ Other, please provide details (can include the strategies provided by the physiotherapist to the informal caregiver) (8) ____

Do you think the person with dementia/cognitive impairment has successfully demonstrate the safe and correct use of gait aid in this session?

- ○ Yes (1)
- ○ Partially (2)
- ○ No (3)

Do you (physiotherapist) have any further comments regarding the gait aid training program?

____

Please provide your (physiotherapist) name so that the research team can contact you in case of queries.

____

Thank you for completing the data collection survey. Please move to the next page to submit the survey online.

End of Block: Demographics--Interview the person with dementia and the caregiver

Appendix J

Gait aid-training for people with dementia--6th week (fourth home visit)

Start of Block: Demographics--Interview the person with dementia and the caregiver

This is the **final assessment (for the participants who you do not recommend use of gait aid after this session)** which occurs at the **fourth home visit** at the 6th week.

You will do the following at this home visit:

- Conduct an assessment for the participants before gait aid training (complete first half of the survey)
- Conduct gait aid training with the person with dementia/cognitive impairment
- Conduct an assessment for the participants after gait aid training (complete second half of survey)
- Make your recommendation for whether the participants will continue to use the gait aid in the longer term or not. If you do not recommend gait aid use in the longer term, remove the gait aid from the participant.
- Collect falls and home practice diary from participants who you do not recommend use of gait aid after this session OR
- Collect home practice diary only from participants who you recommend long term use of gait aid after this session and instruct them to continue filling in their falls diary

This questionnaire includes assessments to be performed **BEFORE and AFTER** the gait aid training session:

Assessments **before** training:

- Number of falls and its associated factors in the last two weeks
- Adverse events in the last two weeks associated with home practice if applicable
- Adherence to home practice sessions carried out by the person with dementia or cognitive impairment and informal caregiver in the last two weeks
- Physical assessments (without a gait aid)

Assessment **after** training:

- Repeat physical assessments (with the recommended gait aid)
- Rate perception of safety and appropriateness of gait aid use by the physiotherapist, the person with dementia or cognitive impairment and the informal caregiver

---

Please enter the ID of the person with dementia or cognitive impairment (using initials and date of birth). For example John Smith, date of birth 14/2/1950 will be JS14021950

____

Record the date of this home visit. For example 3/1/2021 will be 03012021

____

Physiotherapist to answer this question:
Can the person with dementia or cognitive impairment move around their home, including stopping

and turning (observe with gait aid if it has been left with the person, or without if it has not been left with the person)?

- ◯ can do, appears steady (without gait aid) (1)
- ◯ can do but appears unsteady (without gait aid) (2)
- ◯ cannot do without assistance from someone else (without gait aid) (3)
- ◯ can do, appears steady (with gait aid) (4)
- ◯ can do but appears unsteady (with gait aid) (5)
- ◯ cannot do without assistance from someone else (with gait aid) (6)

Can the person with dementia or cognitive impairment move outside their home (self i.e. person with dementia/cognitive impairment or carer report)?

- ◯ can do, reported as steady and safe (without gait aid) (1)
- ◯ can do but reported as unsteady or unsafe (without gait aid) (2)
- ◯ cannot do without assistance from someone else (without gait aid) (3)
- ◯ can do, reported as steady and safe (with gait aid) (4)
- ◯ can do but reported as unsteady or unsafe (with gait aid) (5)
- ◯ cannot do without assistance from someone else (with gait aid) (6)

Can the person with dementia or cognitive impairment walk up and down at least 3 stairs without a handrail or assistance from someone else (self i.e. person with dementia/cognitive impairment or carer report)?

○ can do without difficulty (without gait aid) (1)

○ can do but with difficulty, or needs rail assistance (without gait aid) (2)

○ cannot do without assistance from someone else (without gait aid) (3)

○ can do without difficulty (with gait aid-only score for unilateral gait aid) (4)

○ can do but with difficulty, or needs rail assistance (with gait aid-only score for unilateral gait aid) (5)

○ cannot do without assistance from someone else (with gait aid-only score for unilateral gait aid) (6)

Physiotherapist to answer this question:
Can the person with dementia or cognitive impairment bend and pick up an object from the floor without assistance from someone else (observe)?

○ can do without difficulty (1)

○ can do but with difficulty or unsteady (2)

○ cannot do without assistance from someone else (3)

Did the person with dementia or cognitive impairment fall in the last 2 weeks?
A fall is defined as "inadvertently coming to rest on the ground, floor or other lower level, excluding intentional change in position to rest in furniture, wall or other objects.

Please enter the number of falls i.e. 0 for no falls, 1 for 1 fall etc. Leave blank if unknown/cannot remember.

____

If a fall happened in the last two weeks, please give details in the item below: (put all information into each box provided respectively if there were multiple falls and/or injuries from falls i.e. format: fall 1 circumstance=xxx, fall 2=xxx etc, injury 1=xxx, injury 2=xxx)

- ☐ The circumstance of the fall(s) was: (1) ____

- ☐ The injury from the fall(s) was: enter none if no injury (2) ____

Has the gait aid been left for home practice between sessions?

- ○ Yes (1)
- ○ No (2)

If a gait aid was left for home practice, was the person using the gait aid when they fell?

- ○ Yes. Ask the caregiver if they consider that the gait aid might have contributing to the fall--if yes, please give details (1) ____
- ○ No (2)
- ○ No fall (3)

Did the person with dementia or cognitive impairment and informal caregiver adhere to home practice in the last 2 weeks as per the physiotherapist's recommendations?

- ○ Yes. Full adherence to the frequency and duration of practice (1)
- ○ No. Partial adherence to the frequency and duration of practice, please state the reasons why. (2) ____
- ○ No. No practice at all, please state the reasons why. (3) ____
- ○ No home practice was recommended for the last 2 weeks (4)

Ask the person with dementia or cognitive impairment: How did the person with dementia or cognitive impairment find the home practice session (if home practice was applicable)? Rate on a 0-10 point scale (0=extremely difficult, cannot manage, 10=no difficulties at all, can manage it very well) and provide comments.

____

Ask the informal caregiver: How did the informal caregiver find the home practice session (if home practice was applicable)? Rate on a 0-10 point scale (0=extremely difficult, cannot manage, 10=no difficulties at all, can manage it very well) and provide comments.

____

Before training--Physical assessment: 4 metre walk test

Perform the 4 metre walk test (at a comfortable walking speed) **without a gait aid.** Allow a practice trial before the test.

☐ Time required (in seconds, to one decimal place) (1) ____

☐ Gait speed (metre per second i.e. 4 divided by the time taken) (2) ____

☐ No. of steps (3) ____

☐ Step length (i.e. 4 m divided by the no. of steps) (4) ____

Before training--Physical assessment: Figure of 8 walkway

Perform the Figure of 8 walkway test (at a comfortable speed) **without a gait aid.** Allow a practice trial before the test.

Instructions to the person with dementia or cognitive impairment:

- Stand between the 2 cones (placed 5 feet apart i.e. 1.5 m), facing in one direction (the starting/stopping point is not marked "… in order to avoid influencing the movement planning for the task.")
- Begin walking at your <u>usual pace</u> when ready (the person chooses which direction to go around the cones)
- Stop when you return to the start position.

Instructions to the therapist:

- Timing: begin timing with the first step, and stop timing when the person with dementia assumes a side-by-side stance of the feet back at the start position.
- Steps: count the number of steps taken.
- Accuracy: "The 2-ft (0.6 m) surround test boundary was not marked on the course. The tester determined the 2-ft (0.6 m) boundary area for the test setup and the relationships of the boundary to the testing space (e.g. distance from the hallway walls, floor markings, or

landmarks) prior to testing, and estimated whether the test was completed within the boundary by comparison with the tester's mental map of the testing space."

☐ Time required (in seconds, to one decimal place) (1)
____

☐ No. of steps (2) ____

☐ Accuracy: does the person stay within the unmarked 2-foot (0.6 m) surround boundary of the cones? Write Yes or No response only (3)
____

Before training--repeat the Figure of 8 walkway test (at a comfortable speed) **without a gait aid AND with a dual cognitive task.** Allow a practice trial before the test.

Instructions to the person with dementia or cognitive impairment:

- Stand between the 2 cones (placed 5 feet apart i.e. 1.5 m), facing in one direction (the starting/stopping point is not marked "… in order to avoid influencing the movement planning for the task.")
- Begin walking at your <u>usual pace</u> when ready (the person chooses which direction to go around the cones) and count backwards by 1's from 50 whilst walking.
- Stop when you return to the start position.

Instructions to the therapist:

- Timing: begin timing with the first step, and stop timing when the person with dementia assumes a side-by-side stance of the feet back at the start position.
- Steps: count the number of steps taken.
- Accuracy: "The 2-ft (0.6 m) surround test boundary was not marked on the course. The tester determined the 2-ft (0.6 m) boundary area for the test setup and the relationships of the boundary to the testing space (e.g. distance from the hallway walls, floor markings, or

landmarks) prior to testing, and estimated whether the test was completed within the boundary by comparison with the tester's mental map of the testing space."

☐ Time required (in seconds, to one decimal place) (1) ____

☐ No. of steps (2) ____

☐ Accuracy: does the person stay within the unmarked 2-foot (0.6 m) surround boundary of the cones? Write Yes or No response only. (3) ____

☐ Unable to perform this test (i.e. the person cannot walk and count backwards concurrently- needs to stop) (4)

Perform gait aid training with the participant with dementia/cognitive impairment.
The following questions are to be answered after gait aid training.

What gait aid have you used to provide training for the person with dementia or cognitive impairment?

◯ Walking stick/cane (1)

◯ 4-point stick (2)

◯ Pick-up frame (3)

◯ Wheelie walking frame (4)

◯ Other, please state (5) ____

Has this changed from the previous session?

○ Yes. Please explain why and the factors you considered in changing the gait aid. (1) ____

○ No (2)

After training--Perception rating of safety and appropriateness of gait aid use.

Ask the **participant** with dementia or cognitive impairment after the gait aid training session to rate the following statements:

	Strongly agree (1)	Somewhat agree (2)	Neither agree nor disagree (3)	Somewhat disagree (4)	Strongly disagree (5)
Walking with (state the type of gait aid) has improved my walking (1)	○	○	○	○	○
Walking with (state the type of gait aid) has made me feel safe (2)	○	○	○	○	○
Walking with (state the type of gait aid) feels right for me (3)	○	○	○	○	○

Ask the **participant with dementia or cognitive impairment** to provide comments for the above responses. Record the comments here.

____

Ask the **informal caregiver** after the gait aid training session to rate the following statements:

	Strongly agree (1)	Somewhat agree (2)	Neither agree nor disagree (3)	Somewhat disagree (4)	Strongly disagree (5)
The gait aid has improved the participant's steadiness in walking (1)	○	○	○	○	○
The gait aid has improved the participant's safety in walking (2)	○	○	○	○	○
Using the gait aid is appropriate for the participant (3)	○	○	○	○	○

Ask the **informal caregiver** to provide comments for the above responses. Record the comments here.

____

Give **your opinion** (i.e. physiotherapist) after the gait aid training session to rate the following statements:

	Strongly agree (1)	Somewhat agree (2)	Neither agree nor disagree (3)	Somewhat disagree (4)	Strongly disagree (5)
The gait aid has improved the participant's steadiness in walking (1)	○	○	○	○	○
The gait aid has improved the participant's safety in walking (2)	○	○	○	○	○
Using the gait aid is appropriate for the participant (3)	○	○	○	○	○

Give your (i.e. physiotherapist) comments for the above responses. Record the comments here.

____

After training--Physical assessment: 4 metre walk test

Perform the 4 metre walk test (at a comfortable walking speed) **with the recommended gait aid.** Allow a practice trial before the test.

- [ ] Time required (in seconds, to one decimal place) (1) ____

- [ ] Gait speed (metre per second i.e. 4 divided by the time taken) (2) ____

- [ ] No. of steps (3) ____

- [ ] Step length (i.e. 4 m divided by the no. of steps) (4) ____

After training--Physical assessment: Timed Up and Go (TUG) Test

Perform the TUG test (at a comfortable speed) **with the recommended gait aid.** Allow a practice trial before the test.

Ask the person with dementia or cognitive impairment to stand up from a standard height kitchen chair (46cm), walk three metres at their comfortable speed, turn, and return to the chair and sit down.

- [ ] Time required (in seconds, to one decimal place) (1) ____

After training--Physical assessment: Figure of 8 walkway

Perform the Figure of 8 walkway test (at a comfortable speed) **with the recommended gait aid.** Allow a practice trial before the test.

Instructions to the person with dementia or cognitive impairment:

- Stand between the 2 cones (placed 5 feet apart i.e. 1.5 m), facing in one direction (the starting/stopping point is not marked "… in order to avoid influencing the movement planning for the task.")
- Begin walking at your usual pace when ready (the person chooses which direction to go around the cones)
- Stop when you return to the start position.

Instructions to the therapist:

- Timing: begin timing with the first step, and stop timing when the person with dementia assumes a side-by-side stance of the feet back at the start position.
- Steps: count the number of steps taken.
- Accuracy: "The 2-ft (0.6 m) surround test boundary was not marked on the course. The tester determined the 2-ft (0.6 m) boundary area for the test setup and the relationships of the boundary to the testing space (e.g. distance from the hallway walls, floor markings, or landmarks) prior to testing, and estimated whether the test was completed within the boundary by comparison with the tester's mental map of the testing space."

☐ Time required (in seconds, to one decimal place) (1)
____

☐ No. of steps (2) ____

☐ Accuracy: does the person stay within the unmarked 2-foot (0.6 m) surround boundary of the cones? Write Yes or No response only. (3)
____

After training--repeat the Figure of 8 walkway test (at a comfortable speed) **with the recommended gait aid AND with a dual cognitive task.** Allow a practice trial before the test.

Instructions to the person with dementia or cognitive impairment:

- Stand between the 2 cones (placed 5 feet apart i.e. 1.5 m), facing in one direction (the starting/stopping point is not marked "… in order to avoid influencing the movement planning for the task.")
- Begin walking at your usual pace when ready (the person chooses which direction to go around the cones) and count backwards by 1's from 50 whilst walking.
- Stop when you return to the start position.

Instructions to the therapist:

- Timing: begin timing with the first step, and stop timing when the person with dementia assumes a side-by-side stance of the feet back at the start position.
- Steps: count the number of steps taken.
- Accuracy: "The 2-ft (0.6 m) surround test boundary was not marked on the course. The tester determined the 2-ft (0.6 m) boundary area for the test setup and the relationships of the boundary to the testing space (e.g. distance from the hallway walls, floor markings, or landmarks) prior to testing, and estimated whether the test was completed within the boundary by comparison with the tester's mental map of the testing space."

☐ Time required (in seconds, to one decimal place) (1)
____

☐ No. of steps (2) ____

☐ Accuracy: does the person stay within the unmarked 2-foot (0.6 m) surround boundary of the cones? Write Yes or No response only. (3)
____

☐ Unable to perform this test (i.e. the person cannot walk and count backwards concurrently-needs to stop) (4)

Ask the person with dementia or cognitive impairment: How did the person with dementia or cognitive impairment find the training session with the physiotherapist? Rate on a 0-10 point scale (0=extremely difficult, cannot manage, 10=no difficulties at all, can manage it very well) and provide comments.

____

Ask the informal caregiver:
How did the informal caregiver find the training session the person with dementia or cognitive impairment had with the physiotherapist?
Rate on a 0-10 point scale (0=extremely difficult, cannot manage, 10=no difficulties at all, can manage it very well) and provide comments.

____

What recommendations have you provided to the person with dementia or cognitive impairment and their informal caregiver at the end of the session? (based on the responses of the person with dementia/cognitive impairment and objective gait/mobility measurements)

- ○ Leave the gait aid with the person with dementia and informal caregiver to use in the long term at all times when up and ambulating. (1)
- ○ Leave the gait aid with the person with dementia and informal caregiver to use in the long term but only at some of the time, please state the situations when the person is recommended to use the gait aid. (2) ____
- ○ Remove the gait aid at the end of the session. Do not recommend the person with dementia to use gait aid. (3)

Please explain the strategies that you have used to help the person with dementia/cognitive impairment to learn the safe and correct use of the gait aid during this session (e.g. memory aids, cues, structuring of practice tasks, environment etc) Select all that apply.

- ☐ Constant pattern of practice (1)
- ☐ Constant pattern of instruction (2)
- ☐ Memory aid (3)
- ☐ Verbal cues (4)
- ☐ Visual cues (5)
- ☐ Start with non-complex environment, then progress to more complex environment (6)
- ☐ Avoid dual tasking during initial learning e.g. minimise talking from the person, talking to the person or stop them from walking with the gait aid when they or you want to talk (7)
- ☐ Other, please provide details (can include the strategies provided by the physiotherapist to the informal caregiver) (8) ____

Do you think the person with dementia/cognitive impairment has successfully demonstrate the safe and correct use of gait aid in this session?

- ○ Yes (1)
- ○ Partially (2)
- ○ No (3)

Has this person with dementia or cognitive impairment successfully achieved safe gait aid use at the completion of the 6-week training program?

○ Yes (1)

○ No (2)

Do you think the length of the training program is adequate for the person with dementia/cognitive impairment to learn the safe and correct use of the recommended gait aid?

○ Yes. Please suggest the length of training you think the person actually needed and explain why (including if less than 6 weeks). (1) ____

○ No. Please suggest how much longer (i.e. on top of 6 weeks) you think the person will need and explain why. (2) ____

○ Unsure. Length of the training program is not the primary issue and explain why. (3) ____

Do you think the frequency of home visits is adequate for the person with dementia/cognitive impairment to learn the safe and correct use of the recommended gait aid?

○ Yes. Please explain why. (1) ____

○ No. Please suggest how frequent the home visits will need to be and explain why. (2) ____

○ Unsure. Frequency of home visits is not the primary issue and explain why. (3) ____

Are there any changes to the standard training program you would like to make? (based on the responses of the person with dementia/cognitive impairment and objective gait/mobility measurements)

____

Do you (physiotherapist) have any further comments regarding the gait aid training program?

____

Please provide your (physiotherapist) name so that the research team can contact you in case of queries.

____

Thank you for completing the data collection survey. Please move to the next page to submit the survey online.

End of Block: Demographics--Interview the person with dementia and the caregiver

Appendix K

Gait aid-training for people with dementia--6 weeks follow-up post program

---

Start of Block: Interview the person with dementia and the caregiver

This is the **6 week follow-up assessment** post program for the participants who you recommended to continue using the gait aid after the gait aid training program.

You will do the following at this follow-up:

- Call the participant to ask if they have continued to use the gait aid in the last 6 weeks. If so, arrange for a home visit. If they have stopped using the gait aid completely, then complete the first half of the survey only on the phone (No home visit is required except to collect the items below).
- Collect the gait aid and falls diary from the participant

For the participants who require a home visit:

- No gait aid training is required in this follow-up
- Conduct an assessment with the participants (complete all parts of the survey)
- Discuss with the participants regarding recommendation of long term use of the gait aid or not.
- Collect the falls diary from the participants.

Please enter the ID of the person with dementia or cognitive impairment (using initials and date of birth). For example John Smith, date of birth 14/2/1950 will be JS14021950

____

Record the date of this assessment. For example 3/1/2021 will be 03012021

____

Did the person with dementia or cognitive impairment fall in the last 6 weeks?
A fall is defined as "inadvertently coming to rest on the ground, floor or other lower level, excluding intentional change in position to rest in furniture, wall or other objects.

Please enter the number of falls i.e. 0 for no falls, 1 for 1 fall etc. Leave blank if unknown/cannot remember.

____

If a fall happened in the last 6 weeks, please give details in the item below:(put all information into each box provided respectively if there were multiple falls and/or injuries from falls i.e. format: fall 1 circumstance=xxx, fall 2=xxx etc, injury 1=xxx, injury 2=xxx)

☐ The circumstance of the fall(s) was: (1) ____

☐ The injury from the fall(s) was: enter none if no injury (2) ____

Was the person using the gait aid when they fell?

○ Yes. Ask the caregiver if they thought the gait aid might have contributed to the fall--if yes, please give details (1) ____

○ No (2)

○ No fall (3)

○ No, participant has stopped using the gait aid (4)

What gait aid have you recommended for the person with dementia or cognitive impairment?

- ○ Walking stick/cane (1)
- ○ 4-point stick (2)
- ○ Pick-up frame (3)
- ○ Wheelie walking frame (4)
- ○ Other, please state (5) ____

Has the person with dementia or cognitive impairment continued to use the recommended gait aid?

- ○ Yes. At all times when walking. Continue with the rest of the survey (1)
- ○ Partial use. Only for certain tasks or at times. Please estimate the percentage of the time walking that the gait aid is used and state the reason why the gait aid was only used partially. Continue with the rest of the survey (2) ____
- ○ No. Please explain why. Discontinue the survey. Thank the participant for their time. (3) ____

The rest of this survey are only for participants who have continued to use or partially using the recommended gait aid after the training program

Physical assessment: 4 metre walk test

Perform the 4 metre walk test (at a comfortable walking speed) **with the recommended gait aid.** Allow a practice trial before the test.

- ☐ Time required (in seconds, to one decimal place) (1) ____
- ☐ Gait speed (metre per second i.e. 4 divided by the time taken) (2) ____
- ☐ No. of steps (3) ____
- ☐ Step length (i.e. 4 m divided by the no. of steps) (4) ____

Physical assessment: Timed Up and Go (TUG) Test

Perform the TUG test (at a comfortable speed) **with the recommended gait aid.** Allow a practice trial before the test.

Ask the person with dementia or cognitive impairment to stand up from a standard height kitchen chair (46cm), walk three metres at their comfortable speed, turn, and return to the chair and sit down.

- ☐ Time required (in seconds, to one decimal place) (1) ____

Physical assessment: Figure of 8 walkway

Perform the Figure of 8 walkway test (at a comfortable speed) **with the recommended gait aid.** Allow a practice trial before the test.

Instructions to the person with dementia or cognitive impairment:

- Stand between the 2 cones (placed 5 feet apart i.e. 1.5 m), facing in one direction (the starting/stopping point is not marked "… in order to avoid influencing the movement planning for the task.")
- Begin walking at your <u>usual pace</u> when ready (the person chooses which direction to go around the cones)
- Stop when you return to the start position.

Instructions to the therapist:

- Timing: begin timing with the first step, and stop timing when the person with dementia assumes a side-by-side stance of the feet back at the start position.
- Steps: count the number of steps taken.
- Accuracy: "The 2-ft (0.6 m) surround test boundary was not marked on the course. The tester determined the 2-ft (0.6 m) boundary area for the test setup and the relationships of the boundary to the testing space (e.g. distance from the hallway walls, floor markings, or landmarks) prior to testing, and estimated whether the test was completed within the boundary by comparison with the tester's mental map of the testing space."

☐ Time required (in seconds, to one decimal place) (1)
____

☐ No. of steps (2) ____

☐ Accuracy: does the person stay within the unmarked 2-foot (0.6 m) surround boundary of the cones? Write Yes or No response only. (3)
____

Repeat the Figure of 8 walkway test (at a comfortable speed) **with the recommended gait aid AND with a dual cognitive task.** Allow a practice trial before the test.

Instructions to the person with dementia or **cognitive** impairment:

- Stand between the 2 cones (placed 5 feet apart i.e. 1.5 m), facing in one direction (the starting/stopping point is not marked "… in order to avoid influencing the movement planning for the task.")
- Begin walking at your usual pace when ready (the person chooses which direction to go around the cones) and count backwards by 1's from 50 whilst walking.
- Stop when you return to the start position.

Instructions to the therapist:

- Timing: begin timing with the first step, and stop timing when the person with dementia assumes a side-by-side stance of the feet back at the start position.
- Steps: count the number of steps taken.
- Accuracy: "The 2-ft (0.6 m) surround test boundary was not marked on the course. The tester determined the 2-ft (0.6 m) boundary area for the test setup and the relationships of the boundary to the testing space (e.g. distance from the hallway walls, floor markings, or landmarks) prior to testing, and estimated whether the test was completed within the boundary by comparison with the tester's mental map of the testing space."

☐ Time required (in seconds, to one decimal place) (1)
____

☐ No. of steps (2) ____

☐ Accuracy: does the person stay within the unmarked 2-foot (0.6 m) surround boundary of the cones? Write Yes or No response only. (3)
____

☐ Unable to perform this test (i.e. the person cannot walk and count backwards concurrently- needs to stop) (4)

Ask the **participant** with dementia or cognitive impairment to rate the following statements:

	Strongly agree (1)	Somewhat agree (2)	Neither agree nor disagree (3)	Somewhat disagree (4)	Strongly disagree (5)
Walking with (state the type of gait aid) has improved my walking (1)	○	○	○	○	○
Walking with (state the type of gait aid) has made me feel safe (2)	○	○	○	○	○
Walking with (state the type of gait aid) feels right for me (3)	○	○	○	○	○

Ask the **participant with dementia or cognitive impairment** to provide comments for the above responses. Record the comments here.

____

Ask the **informal caregiver** to rate the following statements:

	Strongly agree (1)	Somewhat agree (2)	Neither agree nor disagree (3)	Somewhat disagree (4)	Strongly disagree (5)
The gait aid has improved the participant's steadiness in walking (1)	○	○	○	○	○
The gait aid has improved the participant's safety in walking (2)	○	○	○	○	○
Using the gait aid is appropriate for the participant (3)	○	○	○	○	○

Ask the **informal caregiver** to provide comments for the above responses. Record the comments here.

____

Give **your opinion (i.e. physiotherapist)** to rate the following statements:

	Strongly agree (1)	Somewhat agree (2)	Neither agree nor disagree (3)	Somewhat disagree (4)	Strongly disagree (5)
The gait aid has improved the participant's steadiness in walking (1)	○	○	○	○	○
The gait aid has improved the participant's safety in walking (2)	○	○	○	○	○
Using the gait aid is appropriate for the participant (3)	○	○	○	○	○

Give your (i.e. physiotherapist) comments for the above responses. Record the comments here.

____

Do you think the person with dementia/cognitive impairment has successfully retained the skill of safe and correct use of gait aid?

○ Yes (1)

○ Partially. Please state where their skill deficits are present (2) ____

○ No (3)

Discuss the follow-up findings with the person with dementia or cognitive impairment and their caregiver. What recommendations have you provided to them at the end of this follow-up session?

- ◯ Recommend to continue using the gait aid in the long term at all times when up and ambulating (1)
- ◯ Recommend to continue using the gait aid in the long term but only at some of the time, please state the situations when the person is recommended to use the gait aid. (2) ____
- ◯ Do not recommend to continue using the gait aid. Please explain why. (3) ____
- ◯ Other. Please state (4) ____

Do you (physiotherapist) have any further comments regarding the gait aid training program?

____

Please provide your (physiotherapist) name so that the research team can contact you in case of queries.

____

Thank you for completing the data collection survey. Please move to the next page to submit the survey online.

End of Block: Interview the person with dementia and the caregiver
